# A Concerted Action of Hepatitis C Virus P7 and Nonstructural Protein 2 Regulates Core Localization at the Endoplasmic Reticulum and Virus Assembly

**DOI:** 10.1371/journal.ppat.1002144

**Published:** 2011-07-21

**Authors:** Bertrand Boson, Ophélia Granio, Ralf Bartenschlager, François-Loïc Cosset

**Affiliations:** 1 Université de Lyon, Lyon, France; 2 INSERM, U758, Lyon, France; 3 Ecole Normale Supérieure de Lyon, Lyon, France; 4 Department of Infectious Diseases, Molecular Virology, University of Heidelberg, Heidelberg, Germany; Johns Hopkins University - Bloomberg School of Public Health, United States of America

## Abstract

Hepatitis C virus (HCV) assembly remains a poorly understood process. Lipid droplets (LDs) are thought to act as platforms for the assembly of viral components. The JFH1 HCV strain replicates and assembles in association with LD-associated membranes, around which viral core protein is predominantly detected. In contrast, despite its intrinsic capacity to localize to LDs when expressed individually, we found that the core protein of the high-titer Jc1 recombinant virus was hardly detected on LDs of cell culture-grown HCV (HCVcc)-infected cells, but was mainly localized at endoplasmic reticulum (ER) membranes where it colocalized with the HCV envelope glycoproteins. Furthermore, high-titer cell culture-adapted JFH1 virus, obtained after long-term culture in Huh7.5 cells, exhibited an ER-localized core in contrast to non-adapted JFH1 virus, strengthening the hypothesis that ER localization of core is required for efficient HCV assembly. Our results further indicate that p7 and NS2 are HCV strain-specific factors that govern the recruitment of core protein from LDs to ER assembly sites. Indeed, using expression constructs and HCVcc recombinant genomes, we found that p7 is sufficient to induce core localization at the ER, independently of its ion-channel activity. Importantly, the combined expression of JFH1 or Jc1 p7 and NS2 induced the same differential core subcellular localization detected in JFH1- *vs.* Jc1-infected cells. Finally, results obtained by expressing p7-NS2 chimeras between either virus type indicated that compatibilities between the p7 and the first NS2 trans-membrane domains is required to induce core-ER localization and assembly of extra- and intra-cellular infectious viral particles. In conclusion, we identified p7 and NS2 as key determinants governing the subcellular localization of HCV core to LDs *vs.* ER and required for initiation of the early steps of virus assembly.

## Author Summary

Hepatitis C virus (HCV), an enveloped virus that causes chronic liver infection, encodes a polyprotein that is translated and undergoes maturation by cleavage at the endoplasmic reticulum (ER). The assembly of the viral structural components, including core, the capsid protein, the E1/E2 envelope glycoproteins, and the vRNA is believed to occur at the ER, requiring a coordinated integration of cellular and viral pathways in which the HCV non-structural proteins play a major role. The cytosolic lipid droplets (LDs) induce concentration of core close to the ER-located assembly site and may provide a physical link with the vRNA replication site, also localized in specialized, ER-derived structures. Here, we analyzed the subcellular localization pattern of core protein in HCV-infected cells with a particular focus on core colocalization with E2 in the ER or with specific markers of the LDs. We show that the p7 and NS2 proteins are key viral determinants governing the cellular localization of HCV core to LDs *vs.* ER and are required for virus assembly. Our results also underscore a requirement for compatibilities between the p7 trans-membranes and the NS2 amino-terminus that dictates core-E2 colocalization in the ER, leading to initiation of virion assembly.

## Introduction

About 170 million people worldwide are infected with the hepatitis C virus (HCV), a pathogen that causes chronic liver infection often leading to cirrhosis and hepatocellular carcinoma. HCV is an enveloped virus belonging to the genus *Hepacivirus* within the *Flaviviridae* family [1]. The viral genome consists of a single-stranded positive sense RNA molecule of approximately 9.6 kb. It encodes a polyprotein of about 3,000 amino acids that is cleaved both co- and post-translationally at the endoplasmic reticulum (ER) by cellular and viral proteases, giving rise to 10 proteins. The structural proteins include core, the capsid protein, and two envelope glycoproteins, E1 and E2 that mediate binding to co-receptors and entry into hepatocytes [2]–[5]. The non-structural (NS) proteins are separated from the structural proteins by a short membrane protein, p7, thought to act as a viroporin [6]. At least *in vitro*, p7 functions as a calcium ion channel; in cell culture, it is required for virus assembly and optimal release from infected cells [7], [8] by altering the pH equilibration in intracellular vesicles [9]. *In vivo*, p7 is essential for infectivity [10]. The NS region consists of the 6 following proteins: the cysteine autoprotease NS2, the serine protease/helicase complex composed of NS3 and NS4A, two proteins involved in genome replication and assembly, NS4B and NS5A, and the RNA-dependent RNA polymerase NS5B [11].

An essential function of the NS proteins is to generate cellular conditions necessary for i) viral genome replication and mRNA synthesis in specialized, ER-derived structures called replication complexes forming a higher order structure that is known as the membranous web and ii) assembly of viral particles. HCV assembly and envelopment are believed to occur at the ER [12], [13], where E1E2 accumulate [14], [15], and appear to require a coordinated integration of the cellular and viral pathways that bring the viral structural components, core, E1, E2 and viral RNA (vRNA) to the assembly site. Translation of the HCV polyprotein also occurs at ER sites and following maturation by the ER-resident signal peptidase that cleaves core-E1, E1–E2, E2–p7 and p7-NS2, the HCV structural proteins initially remain associated to ER or ER-derived membranes [11], [16]. However, this close vicinity *per se* is not believed to induce assembly of viral particles at ER translation sites since soon after its release from the HCV polyprotein by signal peptide peptidase (SPP) cleavage, the core protein is detected on the surface of lipid droplets (LDs) [15], [17]–[20]. Yet, the degree of core accumulation on LDs appears to depend on particular core sequences and thus the viral isolate [20].

LDs are neutral lipid storage organelles possessing an outer phospholipid monolayer proposed to form by detachment from the cytosolic leaflet of the ER membrane (reviewed in [21]). LDs are mostly tethered to the ER [22] where they serve as a source for lipid esters utilized to generate very low-density lipoproteins (VLDL) in the ER lumen. Transfer of core to LDs requires SPP-mediated removal of a C-terminal fragment corresponding to part of the E1 signal peptide that initially retains core at the ER membrane bilayer [23]. This final maturation event of core is efficient and, at steady state, fully SPP-processed core protein is detected in transfected as well as in cells infected with cell culture-grown HCV (HCVcc) [17]. Core-LD association is mediated by the D2 domain of the core protein, a domain composed of two amphipathic helices and a hydrophobic loop that insert in the LD lipid monolayer [24]. Importantly, some mutants of the D2 domain impaired in transfer to LDs give rise to lower titers of infectious HCV particles [17], [19] thus highlighting the importance of core-LD association in HCV assembly. Progressive accumulation of core on the LD surface occurs within a few hours after infection resulting in complete coating of the organelle in core-expressing cells concomitant with displacement of LD marker proteins, most notably adipophilin-related protein (ADRP) [18]. Core association to LDs is not a cell type specific event as it is observed in most LD-expressing cell types from different species [16], [25]. Whether assembly of HCV particles is restricted to hepatocytes of only humans and chimpanzees remains to be determined.

Until recently, propagation of HCV in tissue culture was not possible. This was overcome by the development of the efficiently replicating full-length HCV genome, of the JFH1 (Japanese fulminant hepatitis clone 1) isolate [26]–[28] and the high-titer Jc1 virus chimera, which is an engineered intra-genotypic chimera between J6-CF and JFH1 HCV strains [29], [30]. The JFH1 HCVcc was shown to replicate and assemble in association with LD-associated membranes, around which core was predominantly detected [17], [19]. However, one study with Jc1 chimeric genomes demonstrated a different binding affinity of core to LDs, suggesting that this difference could be important for efficiency of HCVcc assembly [20].

By comparing the replication of JFH1 and Jc1, we analyzed the subcellular localization pattern of core protein in HCV-infected cells with a particular focus on core colocalization with E2 at the ER or with specific markers of LDs. In particular, we analyzed whether E1–E2, p7 or p7-NS2 proteins expressed in *cis* or in *trans* with core modify its subcellular distribution and we characterized a minimal set of viral proteins as well as their domains involved in JFH1 *vs.* Jc1 differential core subcellular localization and assembly of infectious viral particles.

## Results

### Intracellular core localization at the ER correlates with production of infectious particles

**Figure 1:**
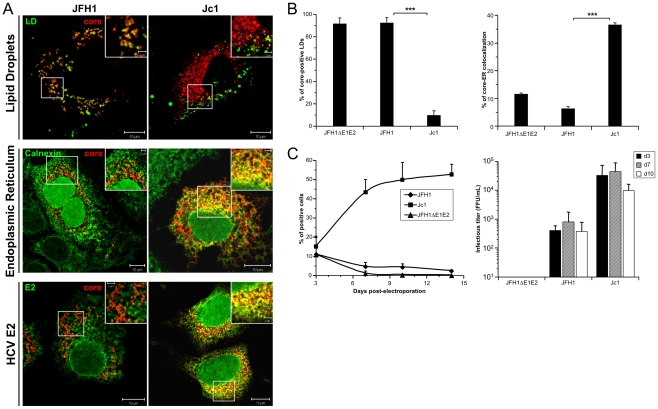
Differential intracellular localization of JFH1 and Jc1 core proteins expressed from HCVcc. Huh7.5 cells were transfected with RNAs from the full-length genomes of JFH1 and Jc1 HCV harboring a nucleus-targeted Venus YFP reporter gene, fixed 72 h post-transfection and stained for LDs, Calnexin, HCV core and E2 proteins. Colocalization of core proteins (red channel) with LD, ER and E2 (green channels) was analyzed by confocal microscopy. Typical patterns of intracellular localization of either protein are shown. The scale bars are provided in each panel as well as in zooms from squared areas. The green fluorescence detected in the nuclei of cells stained with Calnexin and E2 antibodies are those of the nucleus-targeted Venus YFP expressed by the HCVcc. The same differential core-LD *vs.* core-ER localization between JFH1 and Jc1 were detected whether these HCVcc harbored or not this YFP reporter gene (Figure S1B) (**A**). The frequency of JFH1 or Jc1 core-positive LDs (mean % ± SD) was determined in HCVcc-containing cells stained for core and LDs (left panel). The percentages of core-ER colocalization (mean % ± SD) were determined by expressing the coefficients of determination based on Pearson's correlation coefficients of colocalization of core and Calnexin (right panel). For each condition, 30–50 cells were quantified. (*), P<0.05; (**), P<0.01; (***), P<0.001; (ns), no significant difference (**B**). The viral spread in cells expressing JFH1 and Jc1 HCVcc and the JFH1ΔE1E2 negative control was followed by detection of the YFP reporter gene for 14 days (left panel) and the infectious titers (mean ± SD, n = 4) were determined at 3, 7 and 10 days post-transfection (right panel) (**C**).

We investigated the intracellular localization of core and E2 structural proteins in Huh7.5 cells producing JFH1 and Jc1 HCVcc particles using confocal microscopy and subcellular fractionation. Seventy-two hours after transfection with full-length RNA genomes, the core protein showed distinct cellular localization patterns in JFH1- *vs.* Jc1-containing cells (Figure 1). As shown earlier [15], [17]–[20], [23], [24], [31], [32], JFH1 core was mainly detected as ring-like structures associated to LD membranes (*i.e.*, in over 98% of totals LDs; Figure 1B), indicating the accumulation of the core protein on the surface of this organelle (Figure 1A), whereas the E2 glycoprotein was strictly localized at the ER (Figure 1A) and was not detected on LDs. Moreover, JFH1 core protein was poorly detected at the ER (Figure 1A, 1B) in agreement with these previous studies. This was in sharp contrast to Jc1 core that exhibited poor localization on lipids droplets (*i.e.*, on less than 8% of total LDs; Figure 1B), but that was readily detected throughout the ER (Figure 1A, 1B) where it colocalized with E2 (Figure 1A). Identical findings were obtained when fresh Huh7.5 cells were infected with JFH1 and Jc1 HCVcc particles harvested from the supernatants of these Huh7.5 cells 72 hr after transfection (Figure S1A). Likewise, no changes of these differential core intracellular localizations were detected whether the HCVcc carried, or did not carry, a YFP marker gene (Figure S1A *vs.* S1B). Altogether, these results demonstrated that the distinct intracellular core localization patterns observed were intrinsically due to strain-specific features of either virus type and not to transfection-related effects. Finally, similar poor core-LD colocalizations were detected at earlier time points (24 hr and 48 hr) upon infection with Jc1 HCVcc, in contrast to continuous strong core-ER colocalization and to sustained levels of infectious HCVcc production throughout this kinetics (Figure S2).

Interestingly, these different intracellular localization patterns correlated with efficiency of virus production attained with either virus strain. Indeed, as described earlier [20], [30], Jc1 HCVcc exhibited *ca.* 50–100 fold higher infectivity titers than JFH1 (Figure 1C). Furthermore, Jc1 virus was characterized by a rapid propagation in Huh7.5 cells that resulted in infection of 50–60% of cells 10 days post-transfection, whereas JFH1 spread at much lower rates, with a maximum of 5% of infected cells during the same time period (Figure 1C).

**Figure 2:**
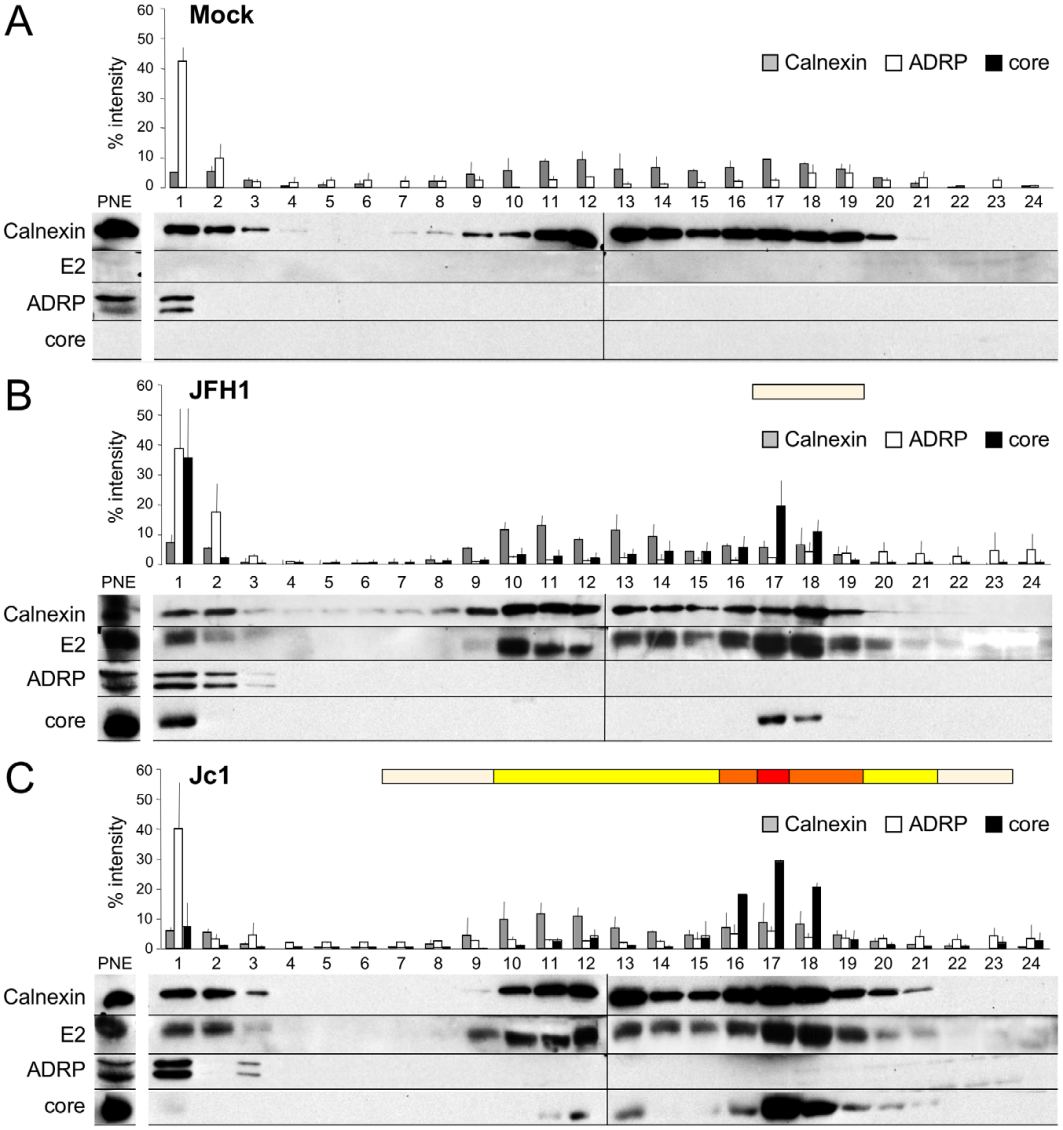
Characterization of JFH1 and Jc1 core localization by subcellular fractionation in HCVcc-expressing cells. Untransfected (**A**) or Huh7.5 cells transfected with RNAs from the full-length genomes of JFH1 (**B**) and Jc1 (**C**) were lysed 72 h post-transfection and *ca.* 2 mg of protein lysates were fractionated on an iodixanol gradient. Each fraction was analyzed by Western blotting using antibodies against Calnexin, ADRP, HCV core and E2 proteins. 1/50 of the unfractionated post-nuclear extracts (PNE) were also analyzed. The infectious titers in each fraction were determined and illustrated as different categories with titers below to 1×10^3^ FFU/fraction (white boxes), from 1 to 3×10^3^ FFU/fraction (yellow boxes), from 3 to 6×10^3^ FFU/fraction (orange boxes), and titers up to 6×10^3^ FFU/fraction (red box).

To confirm that these ER core-E2 colocalization sites represent areas of intracellular HCV assembly, fractionations of JFH1 and Jc1 HCVcc-expressing Huh7.5 cell lysates were performed using gradient centrifugation. We then analyzed the different fractions for infectious, intracellular HCV particles and for core and E2 proteins. LDs and ER present in these fractions were monitored by Western blotting for the markers ADRP (adipophilin-related protein), a LD membrane-associated protein [33], and Calnexin, an ER resident protein (Figure 2). ADRP appeared as a double band, as previously reported [34]. Note that low amounts of Calnexin (and also E2) were also detected in LD-containing fractions, as shown elsewhere [35], [36], and, *vice-versa*, that low amounts of ADRP were detected in the ER fraction, owing to LD tethering to and/or origin from ER membranes [37], [38]. Indeed, in IF studies, neither Calnexin nor E2 were detected on the surface of LDs (data not shown). Interestingly, core showed different enrichment in the subcellular fractions between the two viral clones (Figure 2B and 2C). JFH1 core was mainly observed in top, ADRP-labeled fractions, *i.e.*, fractions 1–3, representing *ca.* 38% of total JFH1 core protein whereas Jc1 core was weakly detected in these LD fractions (less than 9%) but strongly enriched in ER fractions, *i.e.*, fractions 9–21, where E2 co-fractionated (over 85% of total Jc1 core protein). Thus, these results corroborated our observations by confocal microscopy (Figure 1, Figure S1). Furthermore, we found that the intracellular HCV infectivity was detected in ER-containing fractions where core and E2 were detected, but never in LD-containing fractions (Figure 2, see color bars above histograms), thus indicating that core-E2 colocalization in the ER correlates with assembly of infectious HCV particles. Consistently, much lower intracellular infectivity was detected in ER fractions of JFH1 HCVcc-containing cells that produce *ca.* 50–100 fold less infectious particles than Jc1 (Figure 1C).

Altogether, these results indicate that the cellular localization and/or accumulation of core at the ER, which match that of E2, are necessary for efficient assembly and viral particles production.

### ER localization of core in JFH1 HCVcc long-term cultures

**Figure 3:**
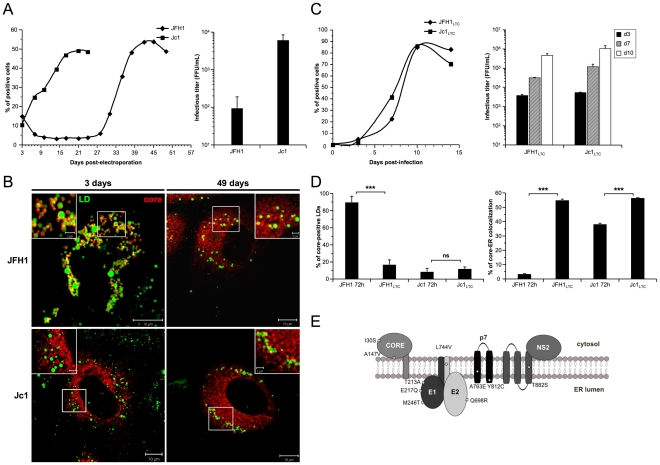
core re-localization in the ER after adaptation of JFH1 HCVcc in long-term culture. Huh7.5 cells were transfected with RNAs from the full-length genomes of JFH1 and Jc1 HCV. The viral spread of the latter viruses was analyzed in these cells culture for up to 49 days (left panel) and the infectious titers, determined as NS5A-FFU/ml (mean ± SD, n = 4) in the supernatants of transfected cells (right panel), were measured 3 days post-transfection (**A**). Cells were fixed at day 3 or day 49 post-transfection and stained for LDs and HCV core. Colocalization of core proteins (red channel) with LDs (green channel) was analyzed by confocal microscopy. Typical patterns of intracellular localization of either protein are shown. The scale bars are provided in each panel as well as in zooms from squared areas (**B**). The supernatants of HCVcc-expressing cells were collected from these cultures at day 3 (JFH1 and Jc1 HCVcc) and at day 49 (JFH1_LTC_ and Jc1_LTC_ HCVcc) and used to infect fresh Huh7.5 cells (MOI = 0.02). The viral spread of the latter viruses was analyzed in these cells culture for up to 15 days (left panel) and the infectious titers, determined as NS5A-FFU/ml (mean ± SD, n = 4) in the supernatants of these cells (right panel), were measured 3, 7 and 10 days post-infections (**C**). JFH1_LTC_ and Jc1_LTC_ HCVcc-infected cells were fixed 72 h post-infection and stained for LDs, Calnexin and HCV core proteins. Colocalization of core proteins with LD or ER was analyzed by confocal microscopy. The frequency of JFH1 or Jc1 core-positive LDs (mean % ± SD) was determined in HCVcc-containing cells stained for core and LDs (left panel). The percentages of core-ER colocalization (mean % ± SD) were determined by expressing the coefficients of determination based on Pearson's correlation coefficients of colocalization of core and Calnexin (right panel). For each condition, 30–50 cells were quantified. (*), P<0.05; (**), P<0.01; (***), P<0.001; (ns), no significant difference (**D**). Schematic representation of residues in HCV proteins that were mutated in core, E1, E2, p7 and NS2 sequences of several JFH1_LTC_ clones isolated. The changes in residues refer to the sequence of the parental JFH1 strain (**E**).

As recent studies have characterized the adaptation of JFH1 and intergenotypic chimeras, resulting in the selection of viruses with enhanced replication [39], [40], we next analyzed the cellular localization of core protein in JFH1 and Jc1 HCVcc long-term cultures (LTC). Jc1 virus production was characterized by rapid kinetics of virus release and spread of infection affecting up to 50% of cells at day 21 (Figure 3A). In contrast, JFH1 HCVcc propagation remained restricted to up to 5% of the cell culture until day 24, when virus spread suddenly increased and reached a maximum of 55% of infected cells at day 43 (Figure 3A, left panel), suggesting an adaptation of virus propagation during LTC, as discussed earlier [41]. Surprisingly, when the cellular localization of core was analyzed in HCVcc-infected LTCs, JFH1 core displayed a predominant ER localization pattern at day 49, with some remaining associations to LDs, *i.e.*, in *ca.* 16% of total LDs (Figure 3B). This pattern, which reproducibly appeared in independent HCVcc long-term cultures, was in sharp difference to the strict JFH1 core LD-localization detected at day 3 of the culture, *i.e.*, in over 98% of total LDs (Figure 3B, Figure 1A, 1B, Figure S1). In contrast, the subcellular localization of Jc1 core remained unchanged throughout the culture period of Jc1 HCVcc (Figure 3B) consistent with constantly high virus titers obtained with this chimera. No significant changes in the sizes and numbers of LDs were detected in long-term cultures of JFH1 or Jc1 HCVcc infected cells compared to non-infected cells (data not shown).

Viruses recovered at day 49 from supernatants of JFH1 and Jc1 HCVcc-infected LTCs, termed JFH1_LTC_ and Jc1_LTC_, were then used to infect fresh Huh7.5 cells. Remarkably, JFH1_LTC_ HCVcc propagated as quickly as Jc1 in these cells, in contrast to the parental JFH1 virus (Figure 3C *vs.* 3A, left panels). Furthermore, the infectivity titer of JFH1_LTC_ HCVcc correlated well with the increase of viral spread and the rise in virus titer, by *ca.* 40-fold, between day 3 and day 49 (Figure 3C *vs.* 3A, right panels). Interestingly, this increased infectivity and propagation correlated with localization of core at the ER (Figure 3D, Figure S3), a cellular compartment where E1E2 proteins were detected, and with a loss of core colocalization with LDs (Figure 3D, Figure S3). Altogether, these results indicated that JFH1_LTC_ HCVcc, but not the infected cells themselves, underwent genetic modification(s) that favor(s) spread and infectivity, most likely through sequence changes that optimized assembly of viral particles at the ER. Several mutations were indeed detected along the adapted JFH1_LTC_ HCVcc, in core, E1, E2, p7 and NS2 sequences (Fig. 3E). The investigation of HCVcc genomes harboring these mutations individually or in combination will be reported elsewhere (BB, OG and FLC, in preparation).

### The E1/E2/p7/NS2 polyprotein influence cellular localization of core protein

**Figure 4:**
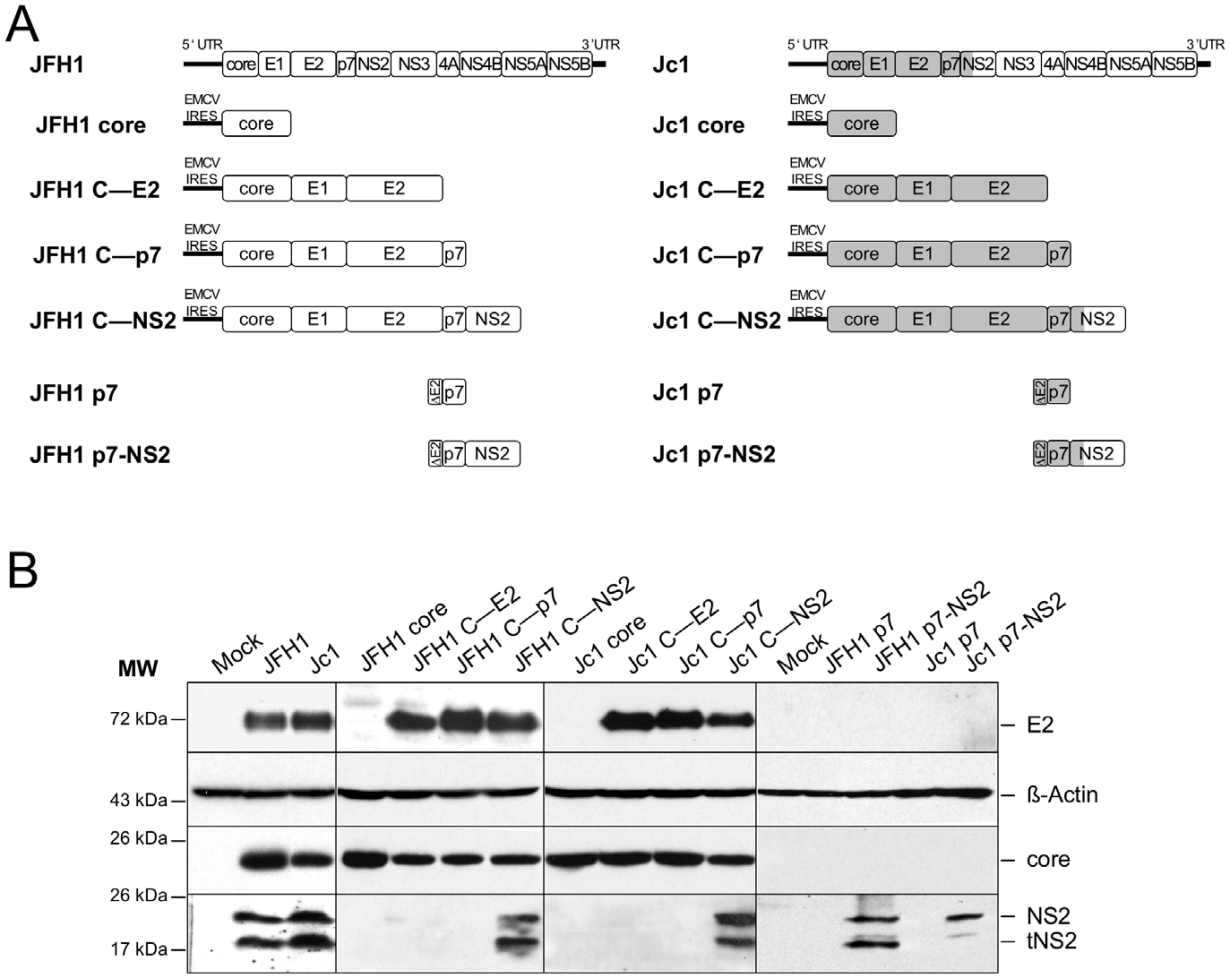
Plasmid constructs and expression levels of core and E2 proteins. Schematic diagrams of HCV proteinexpression constructs used in this study **A**. The sequences encoding core to the first TMS of NS2 derived from the J6CF HCV isolate are indicated in gray boxes whereas sequences from JFH1 strain are shown in white boxes. Core, coreE1E2 CE2, coreE1E2p7 Cp7, coreE1E2p7NS2 CNS2 polyproteins derived from JFH1 and J6CF Jc1 isolates were expressed using a CMV promoter expression construct. The p7 and p7NS2 proteins were expressed using a signal peptide derived from the last 45 aminoacids of HCV E2 E2 *via* transduction with MLVbased retroviral vectors. At 72h posttransfection, lysates from mocktransfected cells, from JFH1 or Jc1 HCVccexpressing cells, from Huh7.5 cells transfected with the JFH1 or Jc1 core, CE2, Cp7 or CNS2 expression constructs, or from Huh7.5 cells transduced with the MLVbased vectors expressing JFH1 or Jc1 p7 or p7NS2, as indicated, were prepared and examined by Western blot analysis using antibodies against NS2, core and E2 proteins **B**. The input of the samples was assessed by staining with an Actin antibody.

To address whether the differential JFH1 *vs.* Jc1 core localization could be influenced by core itself and/or by other HCV factors, we generated a set of constructs that express different HCV proteins, from core to NS2 (Figure 4A). Western blotting analysis demonstrated efficient expression and maturation of core and E2 proteins in Huh7.5 cells transfected with either construct and appropriate cleavage between core and E1, E1 and E2, E2 and p7, and between p7 and NS2 (Figure 4B). A smaller band at *ca.* 17 kDa was detected below NS2 (23 kDa), most likely representing a truncated NS2 form, termed tNS2 as described elsewhere [42], [43].

**Figure 5:**
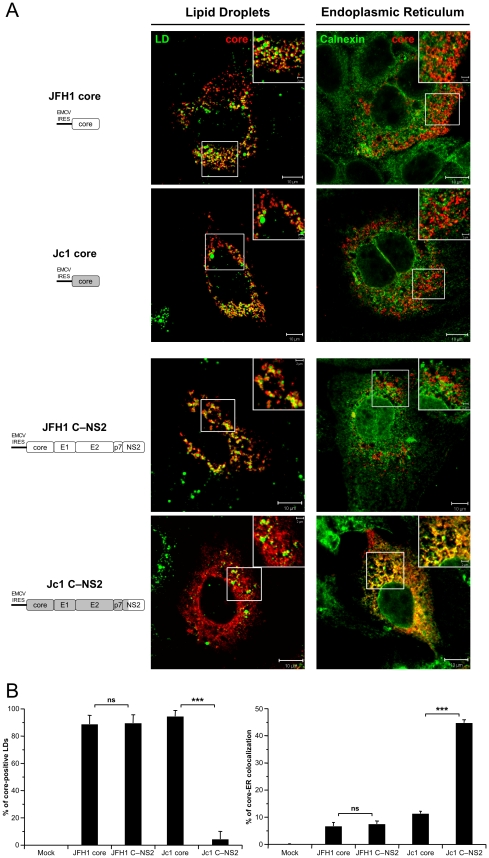
Differential intracellular localization of JFH1 and Jc1 core proteins in cells transfected with core and core—NS2 expression constructs. Huh7.5 cells were transfected with plasmids expressing core alone or core-E1-E2-p7-NS2 (C—NS2) proteins from JFH1 and J6-CF (Jc1) isolates. 72 h post-transfection, cells were stained for LDs, Calnexin, and HCV core proteins. Intracellular localization of core proteins (red channel) in LDs or ER (green channels) was analyzed by confocal microscopy. Typical patterns of intracellular localization of either protein are shown. The scale bars are provided in each panel as well as in zooms from squared areas. The constructs expressed in transfected cells are depicted above each panel (**A**). The frequency of core-positive LDs (mean % ± SD) was determined in HCVcc-containing cells stained for core and LDs (left panel). The percentages of core-ER colocalization (mean % ± SD) were determined by expressing the coefficients of determination based on Pearson's correlation coefficients of colocalization of core and Calnexin (right panel). For each condition, 30–50 cells were quantified. (*), P<0.05; (**), P<0.01; (***), P<0.001; (ns), no significant difference (**B**).

Strikingly, the cellular localization of JFH1 or Jc1 core proteins expressed alone revealed strict localizations to the LDs, *i.e.*, core was detected in up to 95% of total LDs but only very poorly at the ER (Figure 5A, 5B), in sharp contrast to the observations made in HCVcc-containing cells (Figure 1, Figure S1). Since core expressed alone was not secreted in the cells supernatants (data not shown), this indicated that core protein not involved in assembly accumulates on the surface of LDs, therefore arguing for additional viral factors required to target core protein to the ER. Thus, we co-expressed with core the part of the HCV polyprotein sequence differing between JFH1 and Jc1 viruses, *i.e.*, E1, E2, p7, and NS2 [30]. Interestingly, under these conditions, we observed different cellular localization patterns for JFH1 and Jc1 core mimicking those observed in HCVcc-containing cells. Indeed, JFH1 core expressed *in cis* with E1 to NS2 proteins was strictly detected around the LDs, *i.e.*, in *ca.* 90% of total LDs (JFH1 C—NS2 construct), whereas Jc1 core expressed with the Jc1 E1 to NS2 proteins (Jc1 C—NS2 construct) was readily localized at the ER and poorly on the LDs (*i.e.*, in *ca.* 4% of total LDs) (Figure 5A, 5B). Of note and consistent with results obtained with HCVcc-containing cells (Figure 2), upon fractionation of cells expressing core to NS2 polyproteins, JFH1 core was abundantly enriched in ADRP-labeled fractions (*i.e.*, 23% of total JFH1 core protein) in contrast to Jc1 core (*i.e.*, 1%) that was essentially detected in ER fractions (*i.e.*, 84%) (data not shown).

In summary, these results indicate that the co-expression of E1, E2, p7 and/or NS2 with core altered its subcellular localization similar to what was found in cells containing the corresponding full-length genomes. The data further suggest that one, or more, of the HCV proteins affected directly or indirectly the subcellular localization of core.

### Protein p7 induces core localization at the endoplasmic reticulum

As E1E2 glycoproteins accumulate and are retained in the ER (Figure 1A) [14], [44] we first investigated whether E1E2 co-expression with core or core-E1E2 cleavage efficiency between JFH1 and Jc1 strains could modulate the subcellular localization of core. When core and E1E2 proteins were co-expressed (C—E2 constructs) in Huh7.5 cells, the LD localization of core from either strain remained unchanged (*i.e.*, over 95% of LDs were coated whether JFH1 or Jc1 core were co-expressed, or not, with E1E2), as compared to core expressed alone (Figure S4 and data not shown). Likewise, E2 remained associated to the ER compartment whether or not core was co-expressed (data not shown). Thus, these data indicated that core-E1E2 protein co-expression and core-E1E2 cleavage were not implicated in the targeting of core to the LDs *vs.* at the ER.

**Figure 6:**
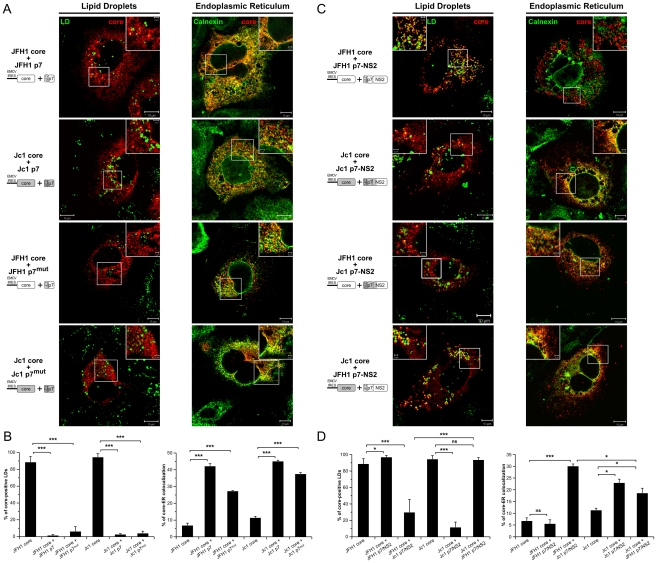
Strain-specific influence of p7 and NS2 on the intracellular localization of core. Huh7.5 cells stably expressing p7 or mutated p7, JFH1-p7^mut^ and Jc1-p7^mut^ (RR33/35AA JFH1-p7 or KR33/35AA Jc1-p7 respectively) proteins (**A**, **B**) and p7-NS2 (**C**, **D**) from JFH1 and J6-CF (Jc1) isolates were transfected with plasmids expressing core from the same HCV strain or from the other isolate. 72 h post-transfection, cells were stained for LDs, Calnexin, and HCV core proteins. Intracellular localization of core proteins (red channel) in LD or ER (green channels) was analyzed by confocal microscopy. Typical patterns of intracellular localization of either protein are shown. The scale bars are provided in each panel as well as in zooms from squared areas. The constructs expressed in transfected cells are depicted above each panel (**A**, **C**). The frequency of core-positive LDs (mean % ± SD) was determined in HCVcc-containing cells stained for core and LDs (left panel). The percentages of core-ER colocalization (mean % ± SD) were determined by expressing the coefficients of determination based on Pearson's correlation coefficients of colocalization of core and Calnexin (right panel). For each condition, 30–50 cells were quantified. (*), P<0.05; (**), P<0.01; (***), P<0.001; (ns), no significant difference (**B**, **D**).

To investigate the potential role of p7 in the subcellular localization of core, we co-expressed in *trans* core and p7 in Huh7.5 cells. Strikingly, co-expression of p7 with JFH1 or Jc1 core resulted in an ER staining pattern as deduced from the strong colocalization of core and Calnexin and in almost absent core-LD colocalization (Figure 6A, 6B). Similar results of core-ER localization were obtained when p7 was expressed stably, *via* retroviral vectors (Figure 6A) *vs.* transiently (data not shown) and when either core or core-E1E2 was expressed along with p7 (compare Figure 6A and Figure S5). Of note, no differences were detected in the distribution, size and number of LDs in cells expressing p7 as compared to mock-transduced cells (Figure S6 and data not shown). Altogether, the data indicate that core expressed alone is intrinsically targeted to the LDs, but the presence of p7, independent of E1E2 and/or cleavage between E2 and p7, induces localization of both JFH1 and Jc1 core at the ER. Moreover, using p7 and core protein sequences from different HCV strains and/or genotypes, *i.e.*, H77, JFH1 and J6-CF, we found that p7-induced core localization at the ER occurred independent from the viral strain/genotype origin of p7 or core and when co-expressing non-autologous core and p7 proteins (data not shown). Finally, when a mutated form of p7 that abolishes its ion-channel function *in vitro* and *in vivo* (RR33/35AA JFH1 p7 or KR33/35AA Jc1 p7) [8], [9] was co-expressed, the core protein remained localized at the ER and was poorly detected on LDs (p7^mut^ in Figure 6A, 6B, and Figure S5).

Altogether, these results indicate that p7 modulates the subcellular localization of core and that this activity is independent of p7 ion channel function; yet, this did not account for the different, strain-specific profiles of core localization observed in cells transfected or infected with full-length HCV genomes (Figure 1, Figure S1), arguing for a specific role of NS2.

### Strain-specific influence of p7-NS2 in cellular localization of core

In order to test the hypothesis of an additional function provided by NS2, we expressed core in the presence of p7-NS2. Like for p7 expressed alone, co-expression of p7-NS2 did not induce differences in size and number of LDs as compared to mock-transduced cells (Figure S6). Interestingly, when core was co-expressed with p7-NS2, Jc1 core localized at the ER and poorly around LDs whereas JFH1 core was only detected around LDs (Figure 6C, 6D). The same differential core localization was detected when core and p7-NS2 were co-expressed with E1E2 (Figure S7). Hence, we concluded that the co-expression of p7-NS2 with core was sufficient to induce the differential subcellular localizations detected in JFH1- *vs.* Jc1-infected cells (Figures 1 and 2, Figure S1A). These results indicated that p7 and NS2 are determinants of core-E1E2 colocalization at the ER.

To determine whether the tripartite relationship between core, p7 and NS2 was strain-specific, we co-expressed JFH1 core with Jc1 p7-NS2 and Jc1 core with JFH1 p7-NS2. Surprisingly, we observed intermediate profiles as compared to the rather strict localization patterns detected for core and p7-NS2 originating from the same HCV strain. Indeed, when co-expressed with non-autologous p7-NS2 constructs, both JFH1 and Jc1 core proteins were readily detected at the ER (Figure 6C, 6D). Yet, a significant proportion of core still remained localized at the surface of LDs (Figure 6C, 6D), although JFH1 core was significantly less often found associated to LDs when co-expressed with Jc1 p7-NS2 as compared to Jc1 core co-expressed with JFH1 p7-NS2.

Altogether, these data indicate that while Jc1 p7-NS2 readily induces localization of core from either virus strain at the ER, there are direct or indirect strain-specific interactions between core, p7 and NS2 that dictate the extent by which core is associated with LDs *vs.* the ER.

### Core-ER colocalization requires compatible trans-membranes in p7 and NS2

**Figure 7:**
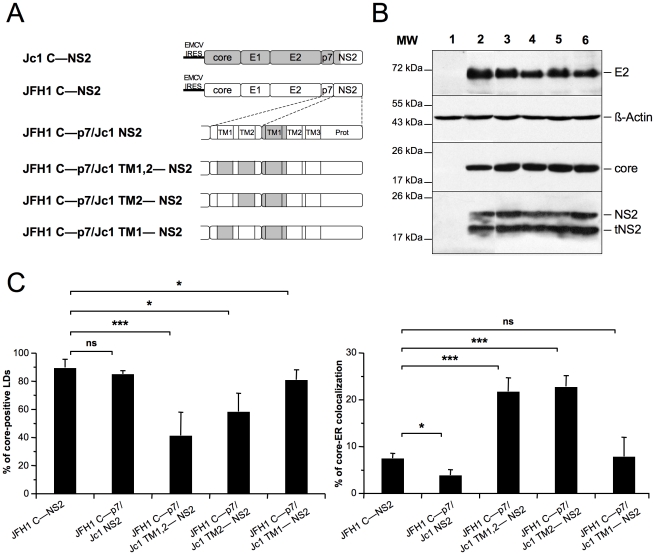
p7 and NS2 trans-membrane compatibility modulates intracellular localization of core. Huh7.5 cells were transfected with plasmids expressing core-E1-E2-p7-NS2 (C—NS2) polyproteins from JFH1 (white boxes) and J6-CF (Jc1) (gray boxes) HCV sequences in which the trans-membrane segments (TM1 and/or TM2) of p7 and/or NS2 were swapped, individually or in combination, as indicated (**A**) Prot, NS2 protease domain. At 72 h post-transfection, lysates from mock-transfected cells (lane 1) or from cells transfected with the JFH1 C—NS2 (lane 2), JFH1 C—p7/Jc1 NS2 (lane 3), JFH1 C—p7/Jc1 TM1,2—NS2 (lane 4), JFH1 C—p7/Jc1 TM2—NS2 (lane 5) and JFH1 C—p7/Jc1 TM1—NS2 (lane 6) expression constructs were prepared and examined by Western blot analysis using antibodies against NS2, core and E2 proteins (**B**). The input of the samples was assessed by staining with an Actin antibody. Cells were stained at 72 h post-transfection for LDs, Calnexin, and HCV core proteins. Intracellular localization of core proteins in LD or ER was analyzed by confocal microscopy. The frequency of core-positive LDs (mean % ± SD) was determined in HCVcc-containing cells stained for core and LDs (left panel). The percentages of core-ER colocalization (mean % ± SD) were determined by expressing the coefficients of determination based on Pearson's correlation coefficients of colocalization of core and Calnexin (right panel). For each condition, 30–50 cells were quantified. (*), P<0.05; (**), P<0.01; (***), P<0.001; (ns), no significant difference (**C**).

To investigate further the molecular basis of core, p7 and NS2 compatibility allowing core-ER *vs.* core-LD colocalization, we designed a series of constructs encoding JFH1 core to NS2 polyproteins in which sub-domains of p7 and/or NS2 were swapped between JFH1 and J6-CF sequences (Figure 7A). All constructs induced similar expression levels of E2, core and NS2 proteins, as compared to the parental constructs (Figure 7B).

Insertion in JFH1 C—NS2 sequence of the first trans-membrane segment (TMS) of J6-CF NS2 [42] (construct JFH1 C—p7/Jc1 NS2, Figure 7A, corresponding to the Jc1 cross-over point [30]), induced core-LD colocalization, but was not sufficient to localize JFH1 core at the ER (Figure 7C). Combined with other results above (Figure 6C, 6D and Figure S7), this suggested that the first TMS of NS2 may require compatibility with p7 to induce core localization at the ER. Indeed, the Jc1 NS2 chimera expressed along with Jc1 p7 was sufficient to localize Jc1 or JFH1 (Figure 6C, 6D) core at the ER. Thus, we expressed this chimeric NS2 protein in the context of JFH1 core to NS2 polyproteins in which the first and/or second TMS of p7 were derived from J6-CF sequence (Figure 7A). We found that replacement of both p7 TMS by those from J6-CF (JFH1 C—p7/Jc1 TM1,2—NS2 construct, Figure 7A) induced core re-localization at the ER and loss from core-LD colocalization (Figure 7C), underscoring the requirement of compatibility between p7 and NS2 TMS for core re-distribution. Furthermore, the replacement of the second p7 TMS (JFH1 C—p7/Jc1 TM2—NS2 construct, Figure 7A) was sufficient to re-localize core at the ER and to reduce LD-localization (Figure 7C). Altogether, these results suggest that a critical interaction and/or compatibility between the second TMS of p7 and the first TMS of NS2 is required to induce core-ER localization.

### HCV assembly and production requires core-ER colocalization induced by p7 and NS2 interactions

**Figure 8:**
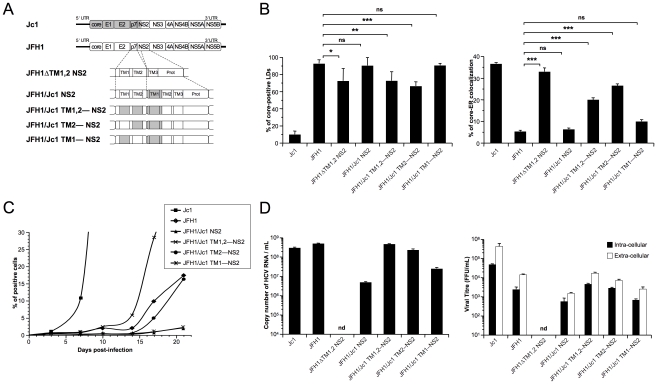
p7 and NS2 trans-membrane compatibility is sufficient to induce production of infectious HCVcc particle. Huh7.5 cells were transfected with full-length RNA derived from the JFH1 genome (white boxes) in which Jc1 sequences (gray boxes), encompassing the trans-membrane segments (TM1 and/or TM2) of p7 and/or NS2, were substituted, or deleted, as indicated (**A**). Prot, NS2 protease domain. Cells were stained 72 h post-transfection for LDs, Calnexin, and HCV core proteins. Intracellular localization of core proteins in LD or ER was analyzed by confocal microscopy. The frequency of JFH1 or Jc1 core-positive LDs (mean % ± SD) was determined in HCVcc-containing cells stained for core and LDs (left panel). The percentages of core-ER colocalization (mean % ± SD) were determined by expressing the coefficients of determination based on Pearson's correlation coefficients of colocalization of core and Calnexin (right panel). For each condition, 30–50 cells were quantified. (*), P<0.05; (**), P<0.01; (***), P<0.001; (ns), no significant difference (**B**). The viral spread in cells infected at MOIs of 0.01 by Jc1, JFH1 and chimeric JFH1 HCVcc was determined for 21 days post-infection, by NS5A immuno-staining (**C**). The copy numbers of HCV RNA (per ml) were determined in the supernatants of HCVcc-expressing cells by RT-qPCR 3 days post-transfection. Jc1 HCVcc input was diluted to 1/100 (left panel). The infectious titers of intra-cellular particles, present in lysates of HCVcc-containing cells, and of extra-cellular particles, present in the supernatants of HCVcc-containing cells, were determined (right panel) as NS5A-FFU/ml (mean ± SD, n = 4). nd, not detectable (**D**).

To address the relevance of these findings in the context of HCVcc assembly, first, we expressed a modified JFH1 genome in which the first and second TMS of NS2 were deleted (JFH1 ΔTM1,2 NS2 construct, Figure 8A). In cells expressing this recombinant, non-infectious HCVcc genome, core protein localized around LDs and was readily detected at the ER, in contrast to the very poor core-ER colocalization detected in cells containing unmodified JFH1 HCVcc (Figure 8B). This phenotype, resembling that of core co-expressed with p7 alone (Figure 6), underscored the conclusion that the loss of a critical p7-NS2 interaction alters core distribution. Thus, we generated a series of JFH1-derived HCVcc recombinant genomes in which the first TMS of NS2 (NS2 TMS1) and either TMS of p7 (p7 TMS1 and p7 TMS2) were substituted, alone or in combination, by those from the J6-CF genome (Figure 8A). Seventy-two hours after transfection of Huh7.5 with full-length RNAs from these genomes and, as control, the parental JFH1 and Jc1 genomes, cells were analyzed for core expression and LD *vs.* ER localization by confocal microscopy (Figure 8B) and for production of infectious HCVcc particles (Figure 8C, D). We found that although core was detected on LDs and/or at the ER, the extent of core localization to each of these compartments differed substantially according to the specific p7/NS2 TMS combination. Interestingly, the levels of core-LD and core-ER associations were similar to those found in cells transfected with the corresponding expression constructs (compare Figure 8 with Figure 7). Overall, these results confirmed that while NS2 TMS1 from J6-CF was not sufficient to induce core localization of JFH1 HCVcc at the ER (JFH1/Jc1 NS2 HCVcc chimera), the combination of both p7 TMS1 and TMS2 with NS2 TMS1 (JFH1/Jc1 TM1,2—NS2 HCVcc chimera) induced core re-localization at the ER (Figure 8B). Moreover, the combination of p7 TMS2 (JFH1/Jc1 TM2—NS2 HCVcc chimera) induced core localization at the ER (Figure 8B).

Importantly, the ER localization of core in cells expressing these HCVcc chimeras correlated with the assembly of infectious particles. First, the introduction of the first TMS from J6-CF NS2 in JFH1 HCVcc (JFH1/Jc1 NS2 chimera; Figure 8A) strongly reduced propagation (Figure 8C) and production and infectivity (Figure 8D) of viral particles, most likely through loss of an interaction between p7 and NS2, in line with a recent study [45]. Second, supporting the hypothesis that matching p7 and NS2 TMS compatibility would restore infectivity, the simultaneous insertion of both J6-CF p7 TMS1 and TMS2 in the latter chimera (JFH1/Jc1 TM1,2—NS2 chimera; Figure 8A) increased production of both infectious and physical particles by *ca.* 11- and 95-fold, respectively, relative to the parental JFH1/Jc1 NS2 recombinant HCVcc genome (Figure 8D). Furthermore, the results highlighted compatibility requirements between NS2 TMS1 and either p7 TMS that correlated well core-ER colocalization to production of infectious HCV particles. Indeed, higher production of viral particles was obtained with the p7 TMS2/NS2 TMS1 combination (JFH1/Jc1 TM2—NS2 chimera), as compared to the p7 TMS1/NS2 TMS1 combination (JFH1/Jc1 TM1—NS2 HCVcc), in agreement with the poorer capacity of the latter virus to induce core-ER localization (Figure 8B). Of note, production of both extra-cellular and intra-cellular infectious particles were increased upon optimization of p7/NS2 TMS compatibility (Figure 8D), indicating that p7-NS2 concerted action regulates assembly of viral particles rather than their morphogenesis and/or egress. Finally, the increase of assembly and production of these HCVcc chimeras stimulated the growth of HCVcc in cell culture (Figure 8C). Indeed, while propagation of JFH1/Jc1 NS2 and JFH1/Jc1 TM1—NS2 HCVcc progressed very slowly, upon infection at low multiplicities of infection (MOIs of 0.01), the JFH1/Jc1 TM1,2—NS2 and JFH1/Jc1 TM2—NS2 viruses displayed much faster propagation rates, in a manner correlated with the extent of core-ER localization.

Altogether, these data indicated that core localization at the ER is necessary to allow virus assembly and requires compatible p7 and NS2 TMS.

## Discussion

Soon after synthesis on ER membranes, the HCV core protein accumulates almost quantitatively on LDs surface in core-expressing cells [16] as well as in JFH1 HCVcc-infected cells [17], [19], [20], [23]. It has been proposed [19] that LDs could induce concentration of core close to the ER-located assembly site of viral particles thus providing a physical link with the vRNA replication site, an HCV-modified area of the ER. This close proximity between replication and assembly sites may facilitate the recruitment of the two HCV nucleocapsid components, core and vRNA, and allows their optimal usage towards assembly of the viral particles at E1E2-containing sites of the ER [46], [47]. LDs themselves cannot therefore be considered as assembly sites but, rather, as factories or platforms facilitating the local concentration of the different viral components in order to induce efficient assembly.

Among the different questions that arise from this model, one is how does core dissociate from the LD surface to reach the cytosolic side of the ER-membrane and the virion-forming vesicle that buds within the ER lumen? Our data sheds light on these events and underscore the crucial role of p7 and NS2 as determinants of core-E1E2 colocalization at the ER. Indeed, our results highlight intrinsic differences between low titer (JFH1) and high titer (Jc1) infectivity-producing HCV genomes regarding the intracellular localization of their core proteins. While JFH1 core is found associated around LDs of infected cells, inducing a perfect coating of these organelles, Jc1 core is poorly detected on LDs but rather, is found primarily distributed at the ER (Figures 1 and 2, Figure S1). Different hypothesis could explain the much stronger Jc1 core-ER colocalization, including: i) a rapid core-LD dissociation and re-transfer of core from the LD back to the ER membrane or to ER-derived assembly sites, ii) a blockage of core-LD association, *i.e.*, through a poor transfer of newly synthesized, SPP-cleaved core from the ER membrane to the LD, or iii) an active capture mechanism of core localized at the border of nascent LDs where core transfers from the ER, before core can coat these organelles, towards assembly sites. Several studies [15]–[20] including those performed in JFH1 HCVcc-infected cells have indicated that accumulation of core protein on LDs occurs quantitatively and is dependent on SPP-cleavage, an efficient process in most cell types, as in our experimental conditions (Figure 4). Thus, it seems plausible that Jc1 core-ER localization is a re-association that occurs by fast dissociation of core from LDs and that promotes efficient Jc1 HCV assembly. However, the two latter hypothesis are also plausible since our kinetics experiments in Jc1 HCVcc-infected cells indicated that core does not accumulate around LDs neither at early time-points following infection, before steady-state production of novel viral particles (Figure S2) nor at later time-points, in contrast to JFH1 [17], [23].

While our data do not argue against a specific role of LDs in HCV assembly, they throw light on the functions of these organelles relatively to the ER during the initial steps of formation of viral particles. Indeed our results indicate that such an ER distribution or re-distribution of Jc1 core is likely important for HCVcc assembly since, as shown in this report, this induces colocalization with the viral surface glycoproteins, which correlates well with *ca.* 50–100 fold higher levels of infectious particles, as compared to JFH1. Conversely, much less frequent JFH1 core-ER localization could be detected, in relation with its lower level formation of infectious HCVcc particles. Furthermore, high-titer JFH1 HCVcc rescued after long-term culture in Huh7.5 cells exhibited an ER-localized core as compared to non-adapted JFH1 virus and to Jc1 virus (Figure 3), strongly strengthening the notion that the ER localization of core plays an important role in HCV assembly.

Data of others indicate that core, expressed alone or in JFH1 HCVcc-infected cells, induces important modifications of the LDs and of their intracellular mobility by excluding ADRP, an LD surface-resident protein, which, in turn, results in LDs accumulation to the perinuclear region [18]. This LD redistribution that occurs in a microtubule-dependent manner and involves the dynein motor protein [18], has been shown to induce their close apposition to the ER membrane [22], [38], partially surrounding the LDs and forming ‘egg-cup’ structures that facilitate transfer of small molecules in the absence of membrane fusion [37]. LD apposition to ER membranes may favor the transfer of replicated RNA to core proteins at the ER replication sites and/or the recruitment of core at assembly sites. However, despite its lower particle production, JFH1 but not Jc1 seems to induce such a modification of LD distribution [18], [20]. This suggests that other events, besides the mere LD-ER apposition, promote genome packaging and/or nucleocapsid assembly. Intriguingly, when expressed alone, the core proteins of both JFH1 and Jc1 have an intrinsic property to reach and accumulate on LDs, as shown in this report and by others [16], [25], [31], and are not detected at the ER. Altogether, these data and our results indicated that there are additional, viral strain-specific factors that govern how either core protein expressed in HCVcc-infected cells could be differentially recruited from the LDs to the ER assembly sites.

We reasoned that the identification of such viral factors would be facilitated using a complementation assay whereby core is co-expressed with other HCV protein candidates. Strikingly, our results indicate that the p7 protein is pivotal for the LD *vs.* ER localization of core. Indeed, p7 expression, independent of its strain-specific origin, exhibited the capacity to induce core localization at the ER.

HCV p7, an integral membrane protein, is a viroporin that has an ion-channel activity *in vitro* [48]–[52] and *in vivo* [9]. It most likely forms hexameric or heptameric complexes [53]–[55] and is primarily localized to the ER [56]–[59]. Deletion of p7 from HCV blocks an early event in virus assembly, before the formation of infectious intracellular particles [7], [20], [60]. Interestingly, our results suggest that the p7 ion-channel activity harbored by its small cytosolic loop is not required for core redistribution, because in our complementation assay, core was similarly re-localized to the ER when co-expressed with wt *vs.* p7 ion channel mutants. Recent evidence indicate that p7 has an proton-selective ion-channel activity *in vivo* and protects the acid pH-sensitive intracellular particles by preventing their acidification while transiting through otherwise acidic intracellular compartments [9]. Thus, our data are in line with these previous evidence indicating that the cytosolic loop of p7 is required for egress of infectious particles rather than for the assembly step itself [7]–[9]. Furthermore our results highlight an additional function for p7 at an early stage of viral assembly. We propose that through this function, p7 could recruit core at the ER by interacting with core itself or, alternatively, could alter core-LD colocalization by modifying LD-ER interactions. This may induce the accumulation of core at ER-derived assembly sites or allow the efficient core transfer from the LD to the ER, respectively, through mechanisms that remain to be determined. The conserved early assembly role of p7 from either BVDV [61], a pestivirus that has not been reported to require LDs for virion assembly, or HCV would rather argue for a direct interaction with core rather than for a indirect effect.

Importantly, although p7 induced core-ER localization in core/p7 co-expression assays, this did not fit with our observations in HCVcc-infected cells that revealed differential core-LD *vs.* core-ER localization according to JFH1 *vs.* Jc1 HCV strain-specificities. Interestingly, our results underscore the role of NS2 as another pivotal factor of HCV assembly. Indeed, co-expression of core with p7 and NS2 induced the same differential localization of core as detected in JFH1 *vs.* Jc1 HCVcc-infected cells. NS2 is a hydrophobic protein homodimer containing three transmembrane domains in its N-terminal part [42], [45] and primarily localizes with membranes of the ER [45], [59], [62]). Besides its cysteine auto-protease activity at the NS2–NS3 junction, the precise function of NS2 remains poorly defined although growing evidence suggests its involvement at an early stage of virus morphogenesis rather than during vRNA replication. Indeed, through analysis of point mutants in HCVcc, NS2 was found essential for production of infectious virus and its protease domain, but not its proteolytic activity, seemed important for this function [7], [42], [63], [64]. Furthermore, the characterization of intergenotypic chimeras has highlighted genetic interactions between the first transmembrane domain of NS2 and upstream sequences, whereas downstream NS2 regions function optimally with other NS proteins [30], [65]. Moreover, through NS2 mutations in HCVcc and analysis of revertant, infectious viruses, second-site changes have been identified in the E1, E2, p7, NS2, NS3 and NS4 sequences [8], [45], [65], [66]. Finally, recent studies identified a role of NS2 factoring the coordination of virus assembly through stable interactions with the E1E2 glycoprotein, p7, NS3–NS4A enzyme complex, and, to a lower degree, NS5A [43], [45], [59], [67]. These results were in agreement with immunofluorescence studies demonstrating colocalization of NS2 with E2 and NS3 at the ER or an ER-derived membrane compartment prior to accumulation in close proximity of LDs [45], [59], suggesting that NS2 recruits these factors to assembly sites at the LD-ER interface. Interestingly, in these analysis performed in H77, Jc1 or JFH1 HCVcc-infected cells [43], [45], [67], core was not detected in co-immunoprecipitation studies with NS2, suggesting that additional events are required to bring core, or the vRNA-core complex, to these NS2 complexes.

The molecular basis of these latter events is unknown currently although several evidence argues for p7-NS2 specific interactions that may control the recruitment of core protein to the assembly site. Indeed, in contrast to the predominant core-LD colocalization detected in JFH1 HCVcc-containing cells, a modified JFH1 HCVcc in which the first and second trans-membrane segments of NS2 were deleted induced core localization at the ER (Figure 8), thus mirroring the phenotype of core co-expressed with p7, in the absence of NS2 (Figure 6). Likewise, also indicating that the loss of a critical p7-NS2 interaction altered core distribution, our results support well an earlier report showing that core accumulates around LDs in cells expressing p7-deleted Jc1 HCVcc in contrast to parental Jc1 virus [20]. A recent study provided biochemical and cell biological evidence for a functional interaction between p7 and NS2, which, consistent with our results, was independent of its ion channel function [68]. Other evidence suggests genetic interactions between core, p7 and NS2 and indicates that the latter proteins can compensate assembly-defective mutations in core protein [46]. By using expression constructs and HCVcc recombinant genomes harboring JFH1/J6-CF p7-NS2 trans-membrane chimeras (Figure 7 and Figure 8), we provide a biochemical support for these recent results. Importantly, our study extends them by underscoring a requirement for compatibilities between the p7 and the first NS2 trans-membranes that regulate core-E2 colocalization at the ER and assembly of both extra- and intra-cellular infectious particles. Thus, it seems possible that through p7-NS2 interactions [59], NS2 modulates the capacity of p7 to induce core accumulation at the ER (Figure 6). NS2 could therefore account for two complementary functions during assembly of viral particles, first, by attracting the envelope glycoproteins at the assembly sites [43], [45], [59], [67] and second, by promoting along with p7 the recruitment of nucleocapsids to such sites. In the case of the JFH1 isolate and compared to Jc1, NS2 seems to prevent the capacity of p7 to induce core-ER accumulation (Figure 6), which could perhaps reflects a mechanism aimed to avoid the over-production of viral particles *in vivo*. Yet, one would expect that optimization of p7-NS2 compatibilities in recombinant JFH1-derived genomes harboring JFH1/Jc1 swaps in p7 and NS2 TMS should exhibit higher viral production (Figure 8). However, in a previous study [20], specific core sequences and/or residues were also found to modulate subcellular distribution of core and its mobility. Indeed, the insertion of the J6-CF core sequence or of its D2 domain into the JFH1 genome reduced core localization around LDs, increased its localization at alternative sites (presumably the ER) and resulted in higher yields of infectivity. Thus, our results are in agreement with this study and, combined with it [20] and with the analyses of revertant viruses [8], [45], [65], [66], indicate that interactions between core, p7 and NS2 modulate core distribution and the early stages of viral particle assembly. This presumably facilitates the encountering between HCV nucleocapsids and envelopes.

## Materials and Methods

### Cell culture and reagents

Huh7.5 and 293T cells were grown in Dulbecco's modified minimal essential medium (DMEM, Invitrogen, France) supplemented with 100 U/ml of penicillin, 100 µg/ml of streptomycin, and 10% fetal bovine serum. 293T cells were used as producer cells for the assembly of pseudotyped viral particles. Huh7.5 cells were used for production of HCVcc and as target cells for infection and transfection assays.

Rabbit antiserum against Calnexin (Sigma Aldrich, France), mouse anti-ADRP (clone AP125, Progen, Heidelberg, Germany), mouse anti-Actin (clone AC74, Sigma, France), mouse anti-core 19D9D6 (kind gift from C. Jolivet, bioMérieux, Lyon, France), rat anti-E2 clone 3/11 (kind gift from J. McKeating, University of Birmingham, UK), mouse anti-NS2 6H6 and mouse anti-NS5A 9E10 (kind gift from C. Rice, Rockefeller University, New York, USA) were used according to the manufacturer's instructions.

### Expression constructs

All nucleotide and amino-acid positions refer to the JFH1 genome (GenBank accession number AB047639). To individually express JFH1 and Jc1 core proteins, the core sequences were amplified by PCR from the molecular clones of JFH1 and Jc1 viruses, an intra-genotypic recombinant between JFH1 and J6-CF sequences (AF177036) from core to up to the first domain of NS2 from and the remaining parts from JFH1 sequences [30], and were cloned into the phCMV-IRES expression plasmid [69]. Similar strategies were used to express core-E1E2 (C—E2 construct) and core-E1E2-p7 (C—p7 construct) polyproteins from JFH1 and Jc1 strains. NS2 sequences were amplified by PCR, sequenced and subcloned into the C—p7 expression plasmids to express the core-E1E2-p7-NS2 (C—NS2 construct) polyprotein from either HCV strains. Then, the amino acids (aa) 818 to 846, 763 to 782, 786 to 808, or 763 to 782 and 786 to 808 from the J6-CF sequence were introduced in the JFH1 C—NS2 construct to replace its p7 or NS2 trans-membrane segments, leading to JFH1 C—p7/Jc1 NS2, JFH1 C—p7/Jc1 TM1—NS2, JFH1 C—p7/Jc1 TM2—NS2 and JFH1 C—p7/Jc1 TM1,2—NS2 expression plasmids, respectively. Recombinant JFH1-derived HCVcc genomes with modified p7 and NS2 trans-membrane segments were generated from these latter constructs by swapping the corresponding p7-NS2 sequences. Finally, p7 and p7-NS2 sequences were PCR-amplified from JFH1 and Jc1 molecular clones, fused to the last 45 amino-acids derived from E2 to provide a signal peptide [60] and subcloned into the CHC murine leukemia virus (MLV)-based retroviral vector also expressing hygromycin as a selective marker [70]. The JFH1 ΔTM1,2 NS2 recombinant genome was constructed by an in-frame deletion of the first two NS2 trans-membrane segments, as described in [42] for the Jc1 virus. Details of primers, subcloning strategies and sequences are available upon request.

### *In vitro* transcription, HCVcc production, titration and viral spread kinetics

To generate infectious HCV RNAs, plasmids pFK-JFH1 or nucleus-targeted Venus YFP-encoding pFKi389-Venus-JFH1, pFK-Jc1 or pFKi389-Venus-Jc1, and pFKi389-Venus-JFH1ΔE1E2 DNAs [8], [26], [30], [71], termed JFH1, Jc1 and JFH1ΔE1E2, respectively, were linearized at the 3′ end by AseI digestion and were treated with Mung Bean nuclease. Purified linearized DNAs were used as template for *in vitro* transcription with the RiboMAX (Promega Corp. USA). *In vitro*-transcribed RNA was delivered to cells by electroporation using Gene Pulser II apparatus (Biorad) in a L3 laboratory, according to European safety regulations, and cells were cultured under standard conditions. Infectivity titers were determined as focus-forming units (FFU)/ml. Huh7.5 cells were infected with different dilutions of culture supernatants containing extra-cellular particles or, alternatively, of lysates of HCVcc-infected cells, containing intracellular particles, that were prepared as described before [72]. Three days post-infection, FFUs were detected by FACS for YFP expression or by colony counting following NS5A immunostaining, as described previously [40]. FFU calculations were based on counts of 1 to 5% YFP or NS5A positive cells, respectively. To assess the kinetics of virus spread, Huh7.5 producer cells were split at different times and analyzed by FACS for detection of YFP reporter gene or by NS5A immuno-staining.

### Quantitative detection of HCV RNA

Viral RNAs were isolated from clarified cell supernatants following Triazol/chloroform extraction and from cells pellet using a QRNeasy mini kit (Qiagen, France) as recommended by the manufacturer. 300 ng of RNA was used for Reverse Transcription (RT) using a iScript TM cDNA Synthesis kit (Biorad, France) following a step reaction of 5 min at 25°C, 30 min at 42°C and 5 min at 85°C. 5 µl of the 1/10 diluted cDNA was used for quantitative PCR (qPCR) with a StepOne Real-Time PCR apparatus (Applied Biosystems, France). HCV-specific qPCR was conducted in duplicate utilizing a FastStart Universal SYBR Green Master (ROX) (Roche, Mannheim, Germany) and the following HCV-specific primers: HCVqS: 5′-CAA GCG CCC TAT CAG GCA GT-3′ and HCVqAS: 5′-CTT CAC GCA GAA AGC GCC TA-3′. Reactions were performed in two stages by using the following conditions: stage 1, 5 min at 95°C (initial denaturation); stage 2, 40 cycles of 15 s at 95°C and 1 min at 60°C (amplification). The amount of HCV RNA was calculated by comparison to serial dilution of a full-length HCV genome encoding plasmid.

### Generation of Huh7.5 stably expressing p7 and p7-NS2 proteins

Retroviral vectors expressing p7 and p7-NS2 from JFH1 and Jc1 viruses were produced from 293T cells *via* VSV-G-pseudotyped particles, as described previously [73], [74]. Stable expression of p7 and p7-NS2 in Huh7.5 targets cells was obtained by transduction with retroviral vector-particles recovered from supernatants of 293T producer cells, followed by hygromycin-selection.

### Immuno-fluorescence (IF) and confocal microscopy imaging

Huh7.5 cells were grown on uncoated 14 mm-diameter glass coverslips and transfected using DMRIE-C reagent (Invitrogen, Cergy-Pontoise, France), according to the manufacturer's instructions. The IF stainings were performed 72 h post-transfection at room temperature. The cells were washed with PBS, fixed with 3% of paraformaldehyde in PBS for 15 min, quenched with 50 mM NH4Cl and permeabilized with 0.2% Triton-X-100 for 7 minutes. Subsequently the cells were incubated for 1 h with the primary antibody in 1% BSA/PBS, washed and stained for 1 h with the corresponding fluorescent Alexa-conjugated secondary antibody (Alexa-488 for green channel and Alexa-555 for red channel, Molecular Probes Europe BV, The Netherlands) in 1% BSA/PBS. LD staining was performed using specific cellular tracers of neutral lipids, Bodipy 493/503 (Molecular Probes Europe BV, The Netherlands) according to the manufacturer's instructions. The cells were washed several times with PBS and mounted in Mowiol 40–88 (Fluka, Buchs, Switzerland) prior to image acquisition with LSM 510 confocal equipped with an Axiovert 100 M camera (Carl Zeiss Inc., Thornwood NY, USA). We verified the absence of signal overlap in the red channel as reported in some studies [75]. We also compared permeabilization with Triton-X-100 *vs.* 0.1% Saponin and Mowiol 40–88 *vs.* Fluoromount (Sigma-Aldrich) mounting media for imaging studies of LDs owing to previous studies reporting eventual loss of LD-associated proteins while using detergent such as Triton-X-100 and glycerol-containing mounting media [76], [77]. Using either procedure, we found no differences neither in LD number, size and cellular distribution in mock-infected cells nor in core-LD *vs.* core-ER differential distribution between JFH1 and Jc1 HCVcc-expressing cells (Figure S8). Therefore, the results of IF presented in this article were generated using Triton-X-100 permeabilization and Mowiol 40–88 mounting media.

The frequency of core-positive LDs was determined in cells stained for core and LDs by manual counting of *ca.* 4,000–10,000 LDs. LDs were scored as core-positive when full core-coating of LD was detected, as previously reported [20]. The degree of localization of core at the ER was quantified by determining Pearson's correlation coefficients providing a measure for the relative degree of colocalization of core and Calnexin by using the ImageJ software [45]. The percentage of core-ER colocalization was then determined by expressing the coefficient of determination (square of Pearson's correlation coefficient), which figures the fraction of variability in the green channel that can be explained by its linear regression with the red channel [78].

### Subcellular fractionation

Separation of different membrane compartments was achieved as described previously [79] with some modifications. The Huh7.5 cell pellets were washed in PBS and homogenized in 1 volume of 10 mM Hepes-NaOH 10 mM pH 7.8 (hypo-osmotic buffer). The cells were allowed to swell on ice for 10 min and were re-isolated by centrifugation at 800×g at 4°C for 2 min. The medium was returned to iso-osmoticity by removing 2/3^rd^ of the volume of the supernatant and adding 1/3 volume of 0.60 M sucrose, 10 mM Hepes-NaOH at pH 7.8 (hyper-osmotic buffer). Cells were disrupted by passaging 20 times through a 25 G needle and lysates were separated from the nuclei by centrifugation at 13,000×g for 30 min at 4°C. Subcellular fractionation was performed in three-step iodixanol gradients. Equal protein amounts of the post-nuclear extracts (PNE) were mixed with 60% iodixanol to give a final concentration of 30%, the hypo-osmotic buffer was mixed with 60% iodixanol to generate 10 and 20% iodixanol solutions. Equal volumes of these three solutions were layered in SW60Ti centrifuge tubes and centrifuged at 50 krpm for 3 h at 4°C. 25 Fractions were collected from the top and analyzed by Western blotting, proteins were probed with antibodies directed against core, ADRP, and Calnexin.

### Western blotting

After separation by SDS-polyacrylamide gel electrophoresis (PAGE), protein preparations were transferred to nitrocellulose membranes (Optitran BA-S83, Whatman, Dassel, Germany) and revealed with specific Mab, followed by the addition of goat anti-mouse, anti-rat or anti-rabbit immunoglobulin conjugated to peroxydase (Dako A/S, Glostrup, Denmark). The proteins of interest were revealed by enhanced chemiluminescence detection (SuperSignal West Pico Chemiluminescent, Thermo Scientific, Rockford, USA) as recommended by the manufacturer.

### Statistical analysis

Results were expressed as mean ± SEM of n observations, as indicated in the legends of figures. Sets of data were compared with a Student's t test. Differences were considered statistically significant when P<0.05. Symbols used in figures were (*) for P<0.05, (**) for P<0.01, (***) for P<0.001, and (ns) for no significant difference, respectively.

## Supporting Information

Figure S1 **Differential intracellular localization of JFH1 and Jc1 core proteins expressed in HCVcc-infected cells.** Huh7.5 cells were infected with viruses harvested 72 h post-transfection in the supernatants of cells electroporated with RNAs from the full-length genomes of JFH1 and Jc1 HCV harboring a nucleus-targeted Venus YFP reporter gene (**A**) or from the full-length parental genomes of JFH1 and Jc1 HCV devoid of marker gene (**B**). Cells were fixed 72 h post-infection and stained for LDs, Calnexin, HCV core and E2 proteins, as indicated. Co-localization of core proteins (red channel) with LD, ER and E2 (green channels) was analyzed by confocal microscopy. Typical patterns of intracellular localization of either protein are shown. The scale bars are provided in each panel as well as in zooms from squared areas.
(TIF)

Figure S2 **HCV core does not accumulate around LDs at early time points post-infection in Jc1 HCVcc-infected cells.** Huh7.5 cells were infected with Jc1 viruses at an MOI of 0.2. At different time points post-infection, the supernatants of the infected cells were harvested and the infectious titers (NS5A-FFU/ml) were determined (mean ± SD, n = 4). Cells were then stained for LDs, Calnexin, and HCV core proteins. Intracellular localization of core proteins in LD or ER was analyzed by confocal microscopy. The frequency of Jc1 core-positive LDs (mean % ± SD) was determined in HCVcc-containing cells stained for core and LDs (left panel). The percentages of core-ER colocalization (mean % ± SD) were determined by expressing the coefficients of determination based on Pearson's correlation coefficients of colocalization of core and Calnexin (right panel). For each condition, 30–50 cells were quantified.
(TIF)

Figure S3 **ER localization of core in JFH1 HCVcc long-term cultures.** Huh7.5 cells were infected with viruses harvested in the supernatants of cells transfected with RNAs from the full-length genomes of JFH1 and Jc1 HCV after 49 days of culture (JFH1_LTC_ and Jc1_LTC_ HCVcc). Cells were fixed 72 h post-infection and stained for LDs, Calnexin, HCV core and E2 proteins, as indicated. Co-localization of core proteins (red channel) with LD, ER and E2 (green channels) was analyzed by confocal microscopy. Typical patterns of intracellular localization of either protein are shown. The scale bars are provided in each panel as well as in zooms from squared areas.
(TIF)

Figure S4 **E1E2 glycoproteins do not influence core subcellular localization.** Huh7.5 cells were transfected with plasmids expressing core and core-E1-E2 (C—E2) proteins from JFH1 and Jc1 HCV strains. 72 h post-transfection, cells were stained for LDs, Calnexin and HCV core proteins. Intracellular localization of core proteins (red channel) in LD or ER (green channels) was analyzed by confocal microscopy. The scale bars are provided in each panel as well as in zooms from squared areas. The constructs expressed in transfected cells are depicted above each panel.
(TIF)

Figure S5 **p7 co-expressed with C-E2 induces an ER localization of core.** Huh7.5 cells were transfected with plasmids expressing core-E1–E2 (C—E2) proteins in Huh7.5 cells stably expressing p7 or mutated p7 (labeled p7^mut^ : RR33/35AA JFH1-p7 or KR33/35AA Jc1-p7) proteins from JFH1 and Jc1 HCV strains. 72 h post-transfection, cells were stained for LDs and HCV core proteins. Intracellular localization of core proteins (red channel) in LD or ER (green channel) was analyzed by confocal microscopy. The scale bars are provided in each panel as well as in zooms from squared areas. The constructs expressed in transfected cells are depicted above each panel.
(TIF)

Figure S6 **LD number and size are not affected by stable expression of p7 or p7-NS2.** Huh7.5 cells stably expressing p7, p7/NS2 or p7^mut^ proteins from Jc1 strain were stained for LDs and analyzed by confocal microscopy. The numbers of LD per cell and size were quantified by using an automatic measurement program of the ImageJ software.
(TIF)

Figure S7 **p7/NS2 co-expressed with C—E2 induces a differential localization of JFH1 vs Jc1 core.** Huh7.5 cells were transfected with plasmids expressing core-E1–E2 (C—E2) proteins in Huh7.5 cells stably expressing p7-NS2 from JFH1 and Jc1 HCV strains. 72 h post-transfection, cells were stained for LDs, Calnexin, and HCV core proteins. Intracellular localization of core proteins (red channel) in LD or ER (green channels) was analyzed by confocal microscopy. The scale bars are provided in each panel as well as in zooms from squared areas. The constructs expressed in transfected cells are depicted above each panel.
(TIF)

Figure S8 **Subcellular core localization is not altered by different cell permeabilization and mounting methods.** Huh7.5 cells were mock-transfected (**A**) or were transfected with RNAs from the full-length genomes of JFH1 (**B**) or Jc1 (**C**). 72 h post-transfection, cells were fixed and permeabilized with 0.2% Triton-X-100 or 0.1% Saponin, stained for LDs and HCV core protein, and mounted with Mowiol or Fluoromount, as indicated. Intracellular localization of core proteins (red channel) in LDs (green channels) was analyzed by confocal microscopy. The scale bars are provided in each panel as well as in zooms from squared areas. The numbers of LD per cell and size in mock-transfected cells permeabilized and mounted either with Triton and Mowiol or with Saponin and Fluoromount were quantified by using an automatic measurement program of the ImageJ software (**D**).
(TIF)
