# Supplementary figures and images for: A Concerted Action of Hepatitis C Virus P7 and Nonstructural Protein 2 Regulates Core Localization at the Endoplasmic Reticulum and Virus Assembly
Source: PLoS Pathog. 2011 Jul 21;7(7):e1002144. doi: 10.1371/journal.ppat.1002144 (PMC3141040; doi:10.1371/journal.ppat.1002144)

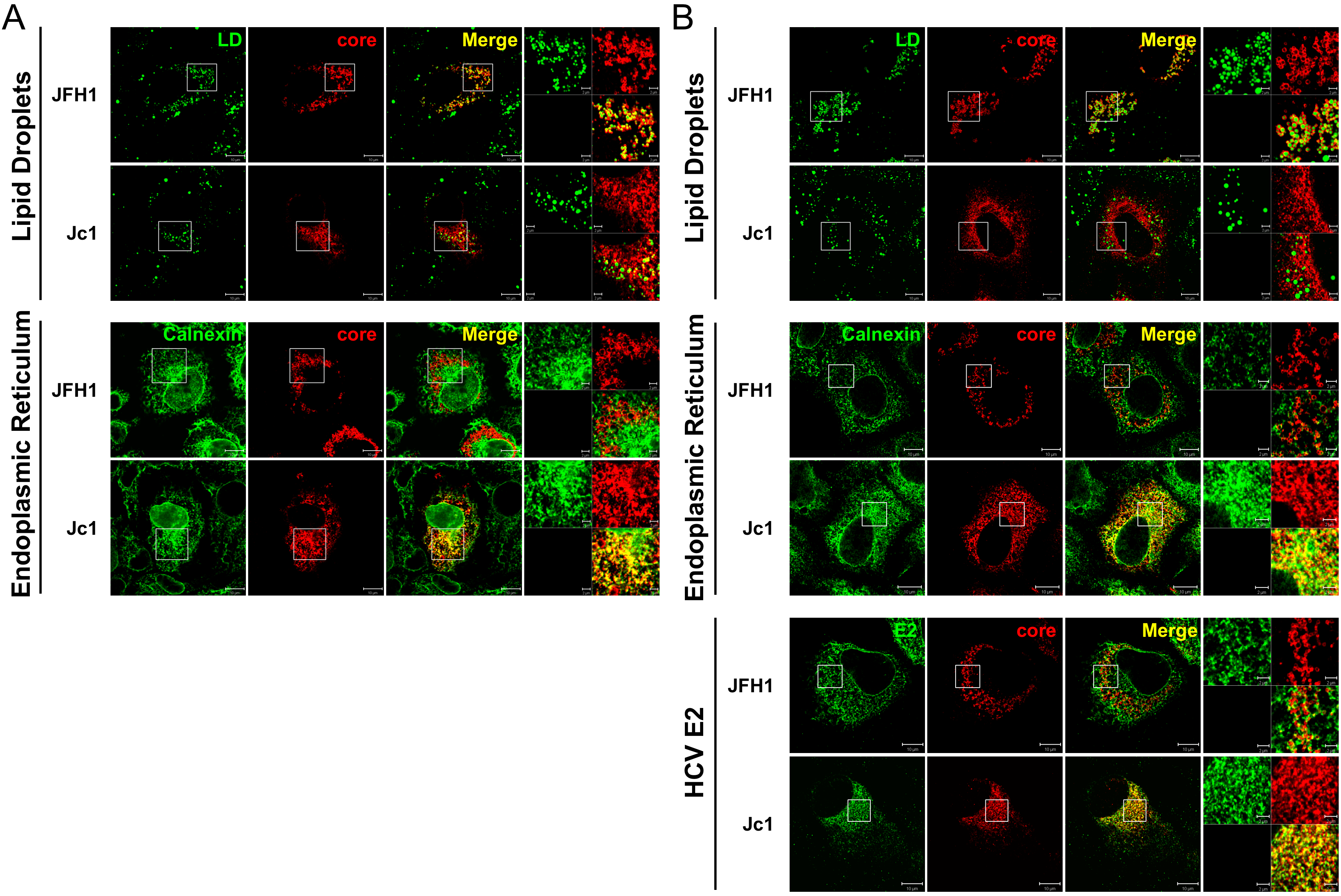

Supplement: Figure S1 — Differential intracellular localization of JFH1 and Jc1 core proteins expressed in HCVcc-infected cells. Huh7.5 cells were infected with viruses harvested 72 h post-transfection in the supernatants of cells electroporated with RNAs from the full-length genomes of JFH1 and Jc1 HCV harboring a nucleus-targeted Venus YFP reporter gene (A) or from the full-length parental genomes of JFH1 and Jc1 HCV devoid of marker gene (B). Cells were fixed 72 h post-infection and stained for LDs, Calnexin, HCV core and E2 proteins, as indicated. Co-localization of core proteins (red channel) with LD, ER and E2 (green channels) was analyzed by confocal microscopy. Typical patterns of intracellular localization of either protein are shown. The scale bars are provided in each panel as well as in zooms from squared areas. (TIF) [file ppat.1002144.s001.tif]

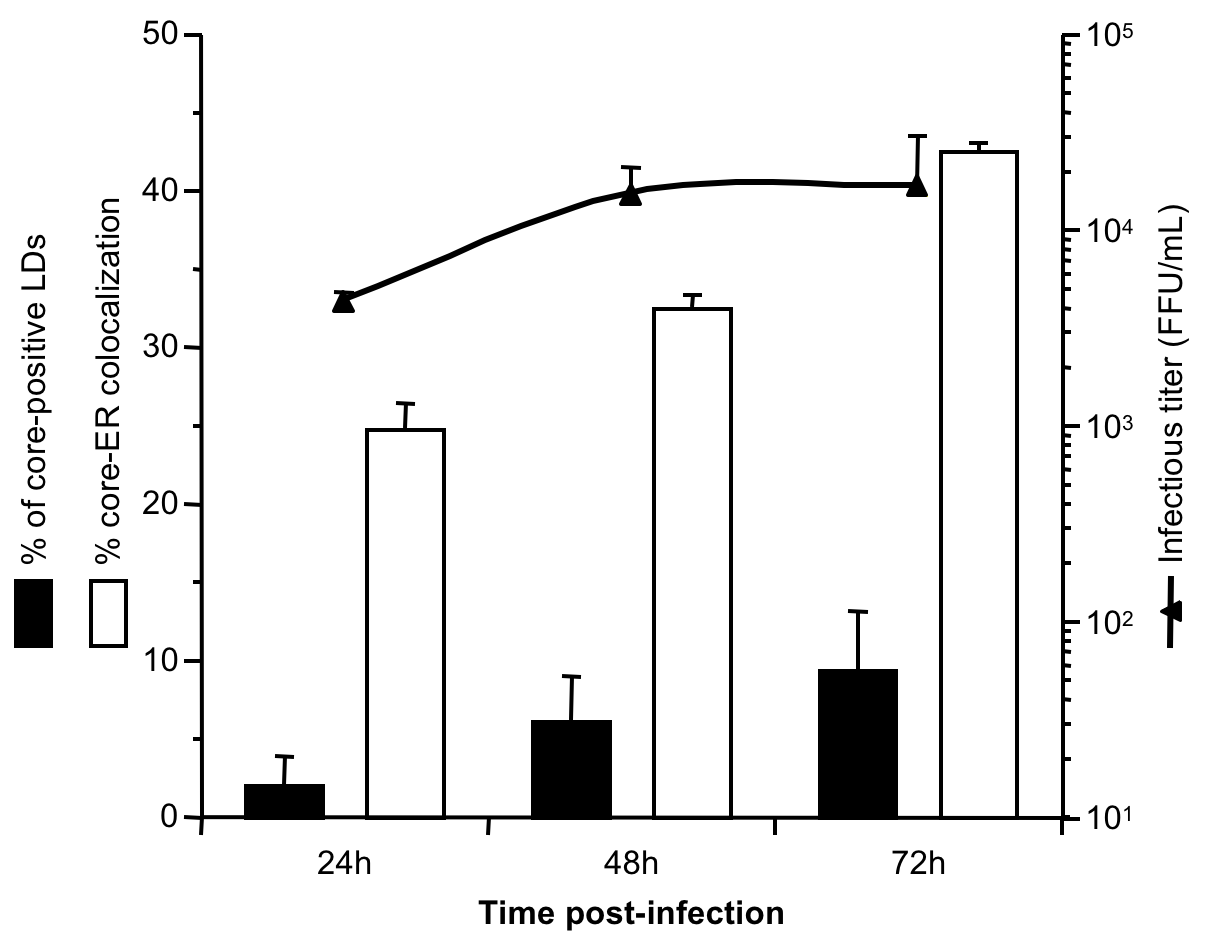

Supplement: Figure S2 — HCV core does not accumulate around LDs at early time points post-infection in Jc1 HCVcc-infected cells. Huh7.5 cells were infected with Jc1 viruses at an MOI of 0.2. At different time points post-infection, the supernatants of the infected cells were harvested and the infectious titers (NS5A-FFU/ml) were determined (mean ± SD, n = 4). Cells were then stained for LDs, Calnexin, and HCV core proteins. Intracellular localization of core proteins in LD or ER was analyzed by confocal microscopy. The frequency of Jc1 core-positive LDs (mean % ± SD) was determined in HCVcc-containing cells stained for core and LDs (left panel). The percentages of core-ER colocalization (mean % ± SD) were determined by expressing the coefficients of determination based on Pearson's correlation coefficients of colocalization of core and Calnexin (right panel). For each condition, 30–50 cells were quantified. (TIF) [file ppat.1002144.s002.tif]

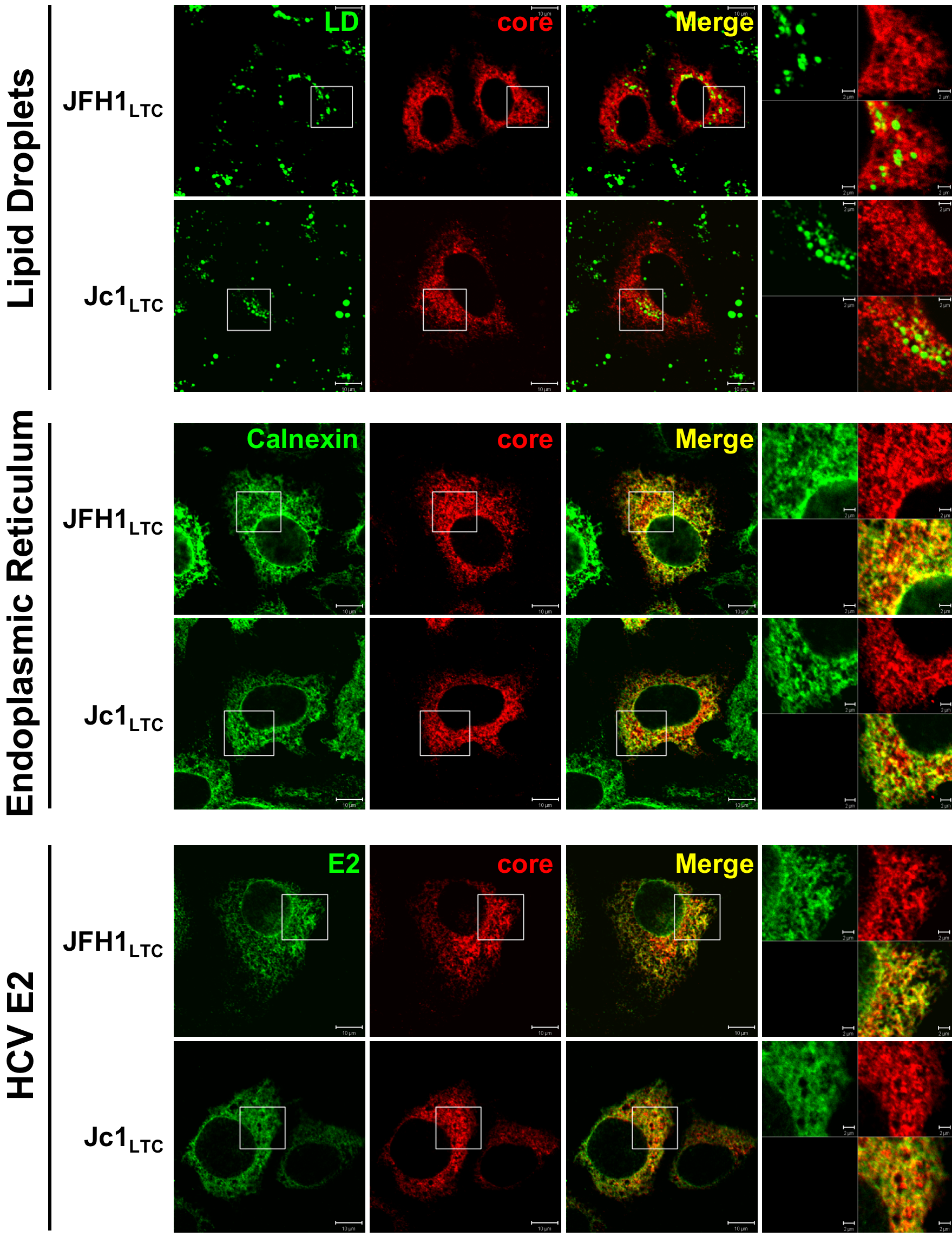

Supplement: Figure S3 — ER localization of core in JFH1 HCVcc long-term cultures. Huh7.5 cells were infected with viruses harvested in the supernatants of cells transfected with RNAs from the full-length genomes of JFH1 and Jc1 HCV after 49 days of culture (JFH1LTC and Jc1LTC HCVcc). Cells were fixed 72 h post-infection and stained for LDs, Calnexin, HCV core and E2 proteins, as indicated. Co-localization of core proteins (red channel) with LD, ER and E2 (green channels) was analyzed by confocal microscopy. Typical patterns of intracellular localization of either protein are shown. The scale bars are provided in each panel as well as in zooms from squared areas. (TIF) [file ppat.1002144.s003.tif]

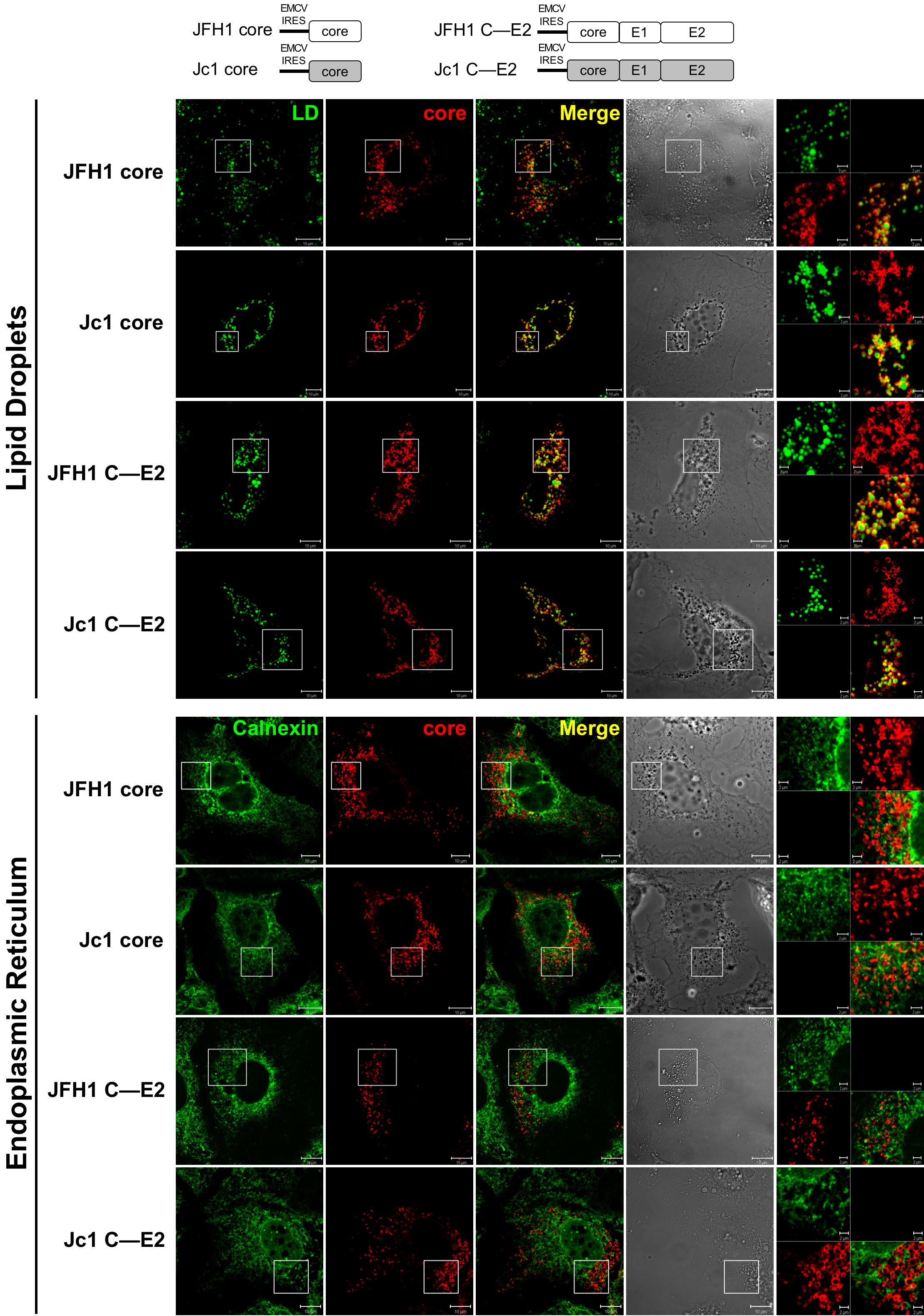

Supplement: Figure S4 — E1E2 glycoproteins do not influence core subcellular localization. Huh7.5 cells were transfected with plasmids expressing core and core-E1-E2 (C—E2) proteins from JFH1 and Jc1 HCV strains. 72 h post-transfection, cells were stained for LDs, Calnexin and HCV core proteins. Intracellular localization of core proteins (red channel) in LD or ER (green channels) was analyzed by confocal microscopy. The scale bars are provided in each panel as well as in zooms from squared areas. The constructs expressed in transfected cells are depicted above each panel. (TIF) [file ppat.1002144.s004.tif]

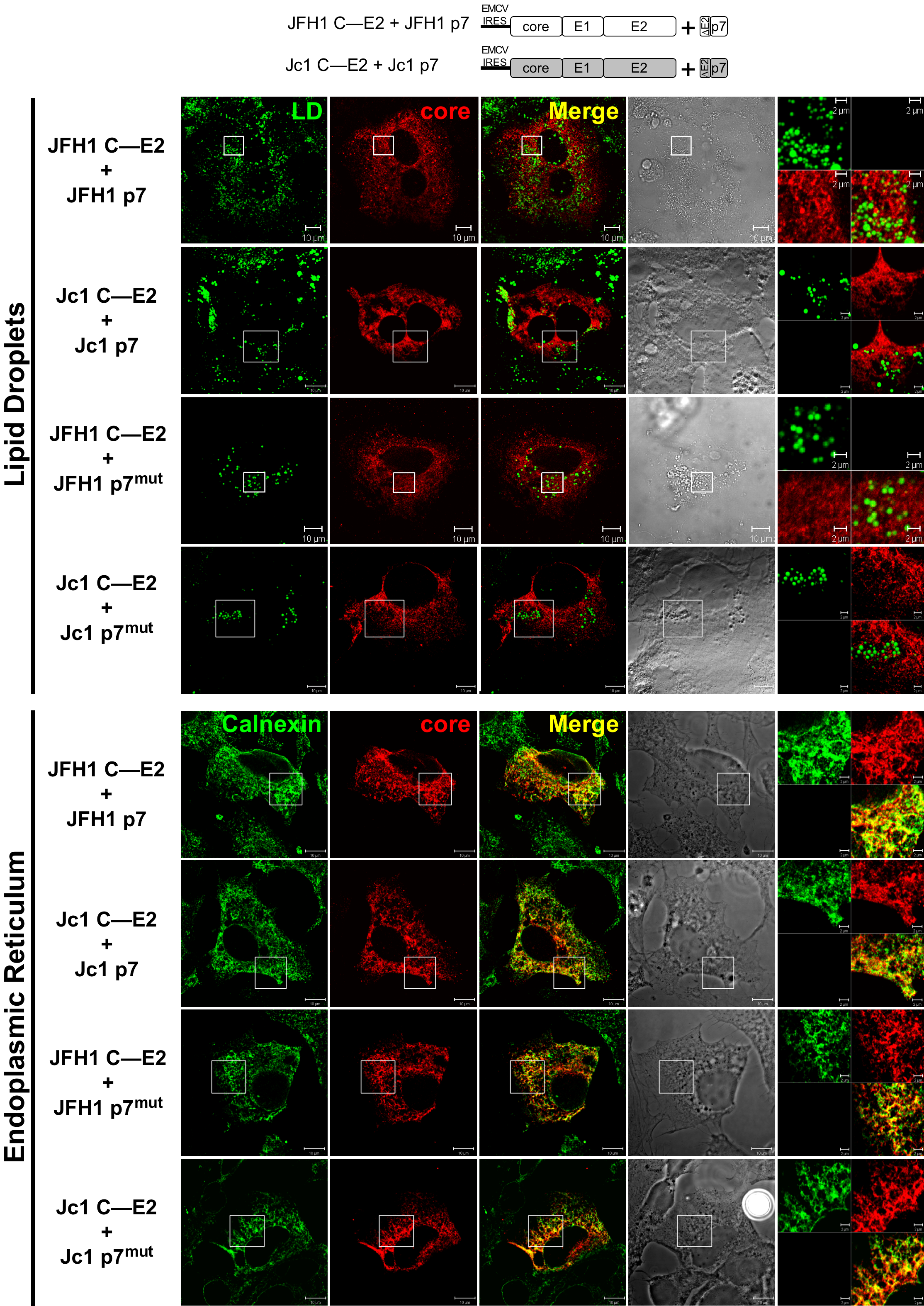

Supplement: Figure S5 — p7 co-expressed with C-E2 induces an ER localization of core. Huh7.5 cells were transfected with plasmids expressing core-E1–E2 (C—E2) proteins in Huh7.5 cells stably expressing p7 or mutated p7 (labeled p7mut : RR33/35AA JFH1-p7 or KR33/35AA Jc1-p7) proteins from JFH1 and Jc1 HCV strains. 72 h post-transfection, cells were stained for LDs and HCV core proteins. Intracellular localization of core proteins (red channel) in LD or ER (green channel) was analyzed by confocal microscopy. The scale bars are provided in each panel as well as in zooms from squared areas. The constructs expressed in transfected cells are depicted above each panel. (TIF) [file ppat.1002144.s005.tif]

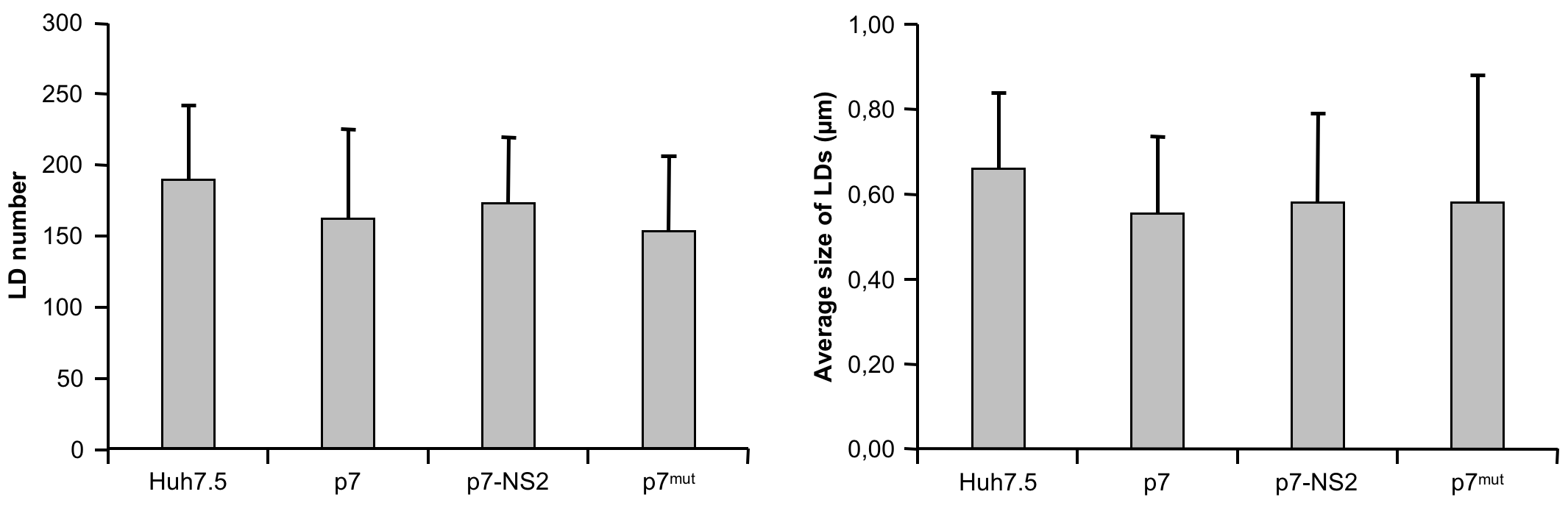

Supplement: Figure S6 — LD number and size are not affected by stable expression of p7 or p7-NS2. Huh7.5 cells stably expressing p7, p7/NS2 or p7mut proteins from Jc1 strain were stained for LDs and analyzed by confocal microscopy. The numbers of LD per cell and size were quantified by using an automatic measurement program of the ImageJ software. (TIF) [file ppat.1002144.s006.tif]

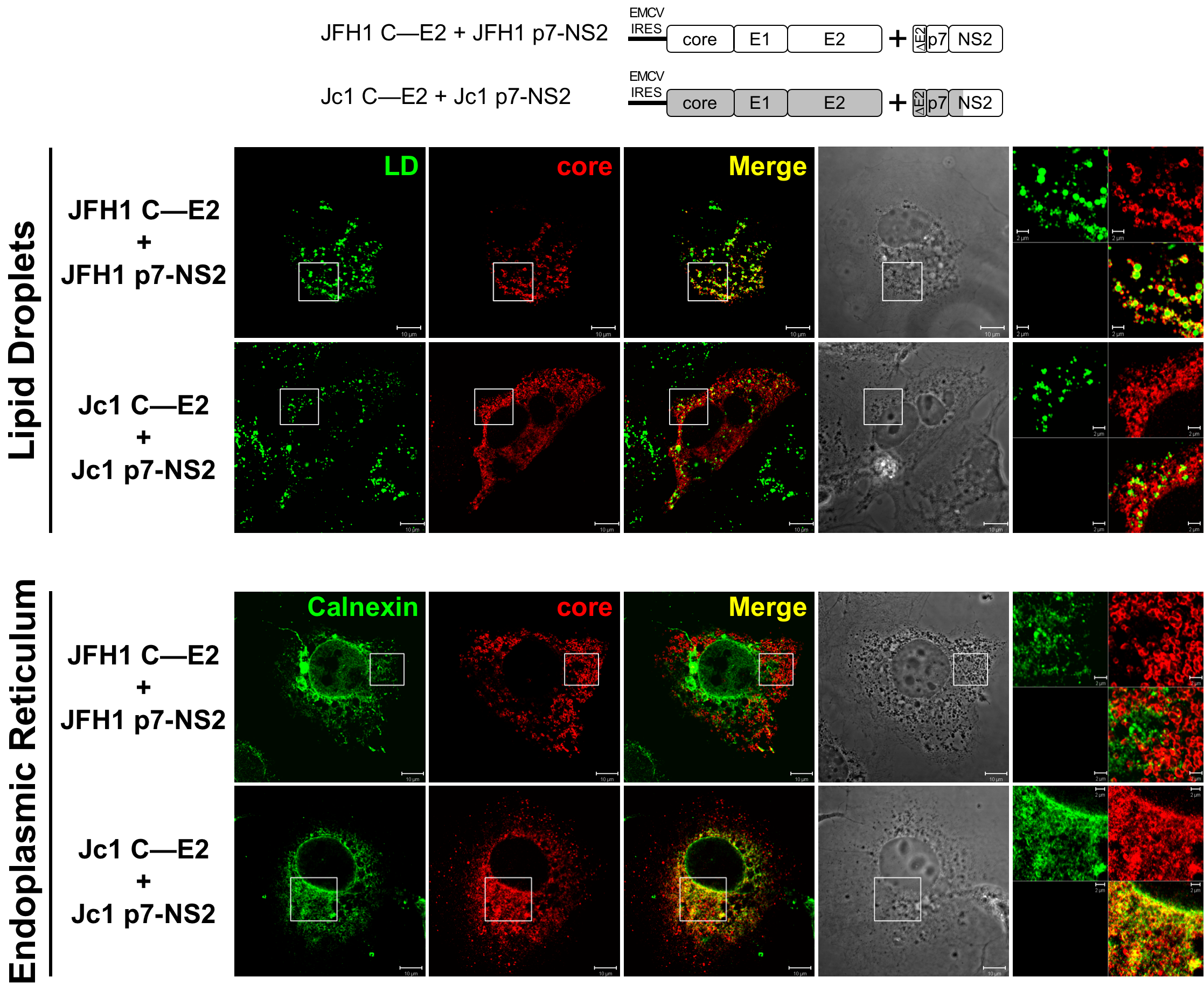

Supplement: Figure S7 — p7/NS2 co-expressed with C—E2 induces a differential localization of JFH1 vs Jc1 core. Huh7.5 cells were transfected with plasmids expressing core-E1–E2 (C—E2) proteins in Huh7.5 cells stably expressing p7-NS2 from JFH1 and Jc1 HCV strains. 72 h post-transfection, cells were stained for LDs, Calnexin, and HCV core proteins. Intracellular localization of core proteins (red channel) in LD or ER (green channels) was analyzed by confocal microscopy. The scale bars are provided in each panel as well as in zooms from squared areas. The constructs expressed in transfected cells are depicted above each panel. (TIF) [file ppat.1002144.s007.tif]

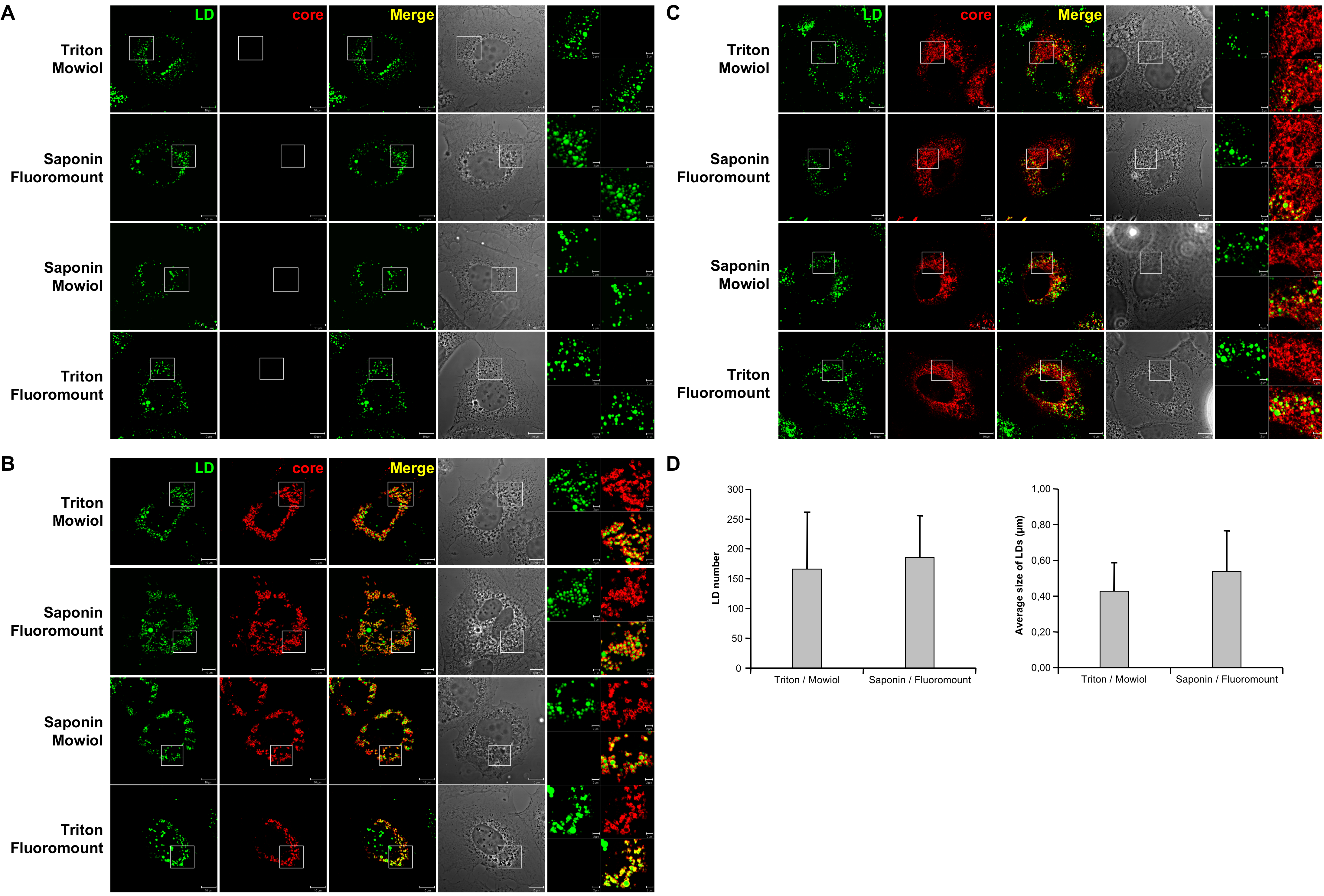

Supplement: Figure S8 — Subcellular core localization is not altered by different cell permeabilization and mounting methods. Huh7.5 cells were mock-transfected (A) or were transfected with RNAs from the full-length genomes of JFH1 (B) or Jc1 (C). 72 h post-transfection, cells were fixed and permeabilized with 0.2% Triton-X-100 or 0.1% Saponin, stained for LDs and HCV core protein, and mounted with Mowiol or Fluoromount, as indicated. Intracellular localization of core proteins (red channel) in LDs (green channels) was analyzed by confocal microscopy. The scale bars are provided in each panel as well as in zooms from squared areas. The numbers of LD per cell and size in mock-transfected cells permeabilized and mounted either with Triton and Mowiol or with Saponin and Fluoromount were quantified by using an automatic measurement program of the ImageJ software (D). (TIF) [file ppat.1002144.s008.tif]
